# ASAS-NANP Symposium: mathematical modeling in animal nutrition: construction of supervised machine learning regression pipelines for livestock data modeling: a case studys

**DOI:** 10.1093/jas/skaf444

**Published:** 2025-12-23

**Authors:** Dan Tulpan, Luis O Tedeschi, Hector Menendez, Ricardo Augusto M Vieira

**Affiliations:** Department of Animal Biosciences, Ontario Agricultural College, University of Guelph, Guelph, ON N1G2W1, Canada; Laboratório de Zootecnia, Universidade Estadual do Norte Fluminense, Campos dos Goytacazes, RJ CEP 28013-602, Brazil; Department of Animal Sciences, Texas A&M University, College Station, TX 77843-2371; Animal Science Department, South Dakota State University, Rapid City, SD 57703; Laboratório de Zootecnia, Universidade Estadual do Norte Fluminense, Campos dos Goytacazes, RJ CEP 28013-602, Brazil

**Keywords:** Animal production, animal science, artificial intelligence, computing, education, machine learning, research, simulation

## Abstract

Integrating open-source tools and machine learning (ML) pipelines into livestock data analysis transforms research, education, and decision-making in animal science. This study presents a comprehensive, end-to-end regression pipeline implemented in Python, designed to predict outcome variables from structured input data in livestock systems. The pipeline includes essential stages of data preparation, such as cleaning, normalization, transformation, and exploratory data analysis, followed by model development, hyperparameter tuning, and interpretability analysis. Two real-world case studies are used to demonstrate the pipeline’s adaptability and predictive capabilities in addressing domain-specific questions in livestock production. The open-source nature of the pipeline serves multiple purposes. First, it promotes reproducibility, a critical requirement in scientific research and data-intensive industry applications, by allowing others to verify and build upon the presented methodology. Second, it enhances accessibility and equity in data science education, enabling students and professionals alike to explore ML applications without the barrier of expensive software or proprietary code. Third, the pipeline is fully modular, encouraging users to adapt, integrate new ML algorithms, and extend components for tasks such as classification, clustering, or time series forecasting in livestock datasets. Beyond its technical implementation, the pipeline emphasizes interpretability, representing an often overlooked yet vital aspect of deploying ML in agricultural contexts. Through the importance of permuted features, residual analysis, and model diagnostics, users gain actionable insights into which variables drive predictions, supporting more informed decisions in herd management, nutrition planning, and breeding programs. This focus ensures that ML outputs are not just accurate, but also meaningful and aligned with real-world livestock production goals. In summary, this work contributes a versatile and transparent machine learning resource tailored for animal science applications. Making the code openly available bridges the gap between methodological advancement and practical deployment, empowering researchers, students, and practitioners to apply ML for better decision-making and scientific discovery in livestock systems.

## Teaser

- Discover how open-source machine learning tools are transforming livestock research and helping predict animal traits with real-world data.
- This flexible Python pipeline empowers researchers, students, and farmers to build accurate, interpretable models for smarter livestock management with no proprietary software required.

## Introduction

Livestock data modeling is crucial to improving animal health, productivity, and farm efficiency. The process typically focuses on capturing the relationship among variables and then applying it to describe and optimize existing systems or predict outcome variable values. The modeling process is typically implemented using a plethora of modeling techniques, including pure mathematical, dynamical, statistical, empirical, or learning-based systems. Perhaps one of the most attractive modeling types in livestock science is building models able to predict variables of interest, such as production or genetic traits, and various statistical and machine learning (**ML**) techniques are available for this purpose. By leveraging machine learning and statistical techniques, farmers can predict disease outbreaks, optimize breeding strategies, enhance overall animal welfare, and optimize the allocation of scarce resources to improve profits. Accurate models help with the early detection of health issues, reducing economic losses and improving sustainability. Additionally, predictive modeling enables better resource management, ensuring optimal feed utilization and reducing environmental impact. As precision livestock farming continues to evolve, data-driven insights will play a key role in shaping the future of animal agriculture.

This study aims to provide a detailed computational and reasoning process to produce a simple and understandable regression-based predictive analytic pipeline that integrates elements of machine learning, statistical mechanisms, and practicality focused on delivering high-quality predictions produced by well-fitted, robust models with an outstanding generalization capability.

## Data Acquisition and Preprocessing for Livestock Modelling

Data acquisition and preprocessing are foundational steps in livestock modeling using machine learning because they directly influence the resulting models’ accuracy, reliability, and applicability. High-quality, well-prepared data ensures that the model can learn meaningful patterns rather than noise or errors, which is especially important given the biological variability in livestock due to genetics, environment, and management practices. Raw data from sensors, video, or farm records often contain missing values, inconsistencies, or noise, which must be addressed through preprocessing to enable robust analysis. Transforming raw inputs into meaningful features through feature engineering enhances model performance and interpretability. Proper preprocessing also helps prevent overfitting and bias by ensuring balanced and standardized datasets.

The importance of data preparation in livestock modeling starts with the “collect and respond” phase, which is often overlooked and is crucial to establish the trustworthiness of data before any analytics is performed (Tedeschi, 2022). That work outlines practical strategies to identify and mitigate issues such as outliers, leverage points, multicollinearity, and violations of distributional assumptions, using tools like DFFITS, Cook’s Distance, variance inflation factors, and robust regression approaches such as Theil-Sen, RANdom SAmple Consensus (RANSAC) or Huber estimators (Tedeschi, 2022; Tedeschi and Galyean, 2024). Visualization techniques like Tukey’s boxplot are powerful, assumption-free tools to uncover unexpected data behavior, but with limitted applicability to smaller datasets. These preprocessing steps serve as a statistical necessity and a strategic advantage in modern animal production systems, where data quality directly informs the capacity to extract actionable insights (Tedeschi, 2022). For significantly larger datasets, traditional statistical outlier detection strategies such as the z-score method are no longer appropriate and data scalable approaches such as clustering (unsupervised machine learning) techniques can be considered (Smiti, 2020).

Ultimately, a strong focus on data acquisition and preprocessing supports the development of scalable, accurate, and practical ML models that can improve animal health, welfare, and farm productivity.

### Sources of livestock data

Livestock data is collected from diverse sources, including wearable sensors and Internet of Things (IoT) devices that track real-time physiological and behavioral factors (Lee and Seo, 2021), such as temperature, activity, and feeding patterns (Neethirajan, 2020; Tedeschi et al., 2021; Chelotti et al., 2024), automated monitoring systems consisting of cameras and computer vision, which analyze movement and interactions among animals (Curti et al., 2023) and manual records obtained via traditional data collection from farm logs and veterinary reports (Görge et al., 2023; Baldin et al., 2025).

### Data cleaning and handling missing values

Data cleaning is crucial in preparing data for machine learning, as it ensures the quality and reliability of the input used to train models. This process involves identifying and handling missing values, correcting errors or inconsistencies, removing duplicates, and standardizing formats (Tedeschi, 2022). Clean data helps prevent biased or inaccurate model outputs and improves overall performance and generalizability. In livestock applications, where data may come from sensors, manual records, or automated systems, cleaning is especially important to address noise, outliers, and irregular sampling (Schodl et al., 2024; Boerman et al., 2025).

Outlier and extreme value detection is also vital in livestock data modeling because such values can significantly distort model training, leading to inaccurate predictions and unreliable insights. Extreme values represent observations or data points that are numerically far from the bulk of the data such as very large or very small relative to the data distribution and represent perfectly valid values, which are expected under the data-generating process. On the other hand, outliers represent observations or data points that do not fit the assumed data-generating process, the expected data distribution or the model being used. These anomalies may result either from sensor errors and data entry mistakes (typically considered outliers) or from rare but genuine biological events (often labeled as extreme values). Proper identification and management, whether through correction, transformation, or removal, helps maintain the integrity of the dataset, ensures model robustness, and supports more accurate and generalizable results. In livestock systems, where variability is natural but data-driven decisions are critical, managing outliers is essential for building trustworthy models. For a more in-depth discussion on identifying and handling outliers in livestock science, refer to the work of Tedeschi (2022).

Moreover, raw livestock data often contains missing values due to sensor malfunctions or data transmission issues. Common techniques for handling missing data include deletion methods that remove incomplete records. However, this may reduce dataset size and introduce bias. An alternative strategy that does not affect dataset size is to use imputation techniques that rely on filling missing values using mean, median, mode, or machine learning model predictions (Little and Rubin, 2019; You et al., 2023), and interpolation and predictive modeling that use regression or deep learning methods to estimate missing values (Donders et al., 2006).

### Feature engineering and feature selection

Feature engineering enhances model performance by creating, transforming or encoding variables (features) from raw data into meaningful features to better represent the underlying problem to the model. Feature engineering techniques include a plethora of approaches such as: 1) numerical transformations (e.g., scaling, normalization, binning) to improve distributional properties or capture nonlinear relationships, 2) encoding of categorical features (e.g., One-hot encoding, label encoding, target encoding, embeddings) to convert categorical variables into numerical representations, 3) feature construction/combination/aggregation that creates new features from existing ones to capture domain knowledge by applying mathematical functions and operators, 4) text manipulations (e.g., Bag-of-Words, TF-IDF, N-grams, word embeddings) to represent unstructured text as numerical features, 5) time series and sequential feature engineering (e.g., lag features for past-to-present predictions, rolling statistics, seasonality indicators, frequency-domain features such as Fourier or wavelet transforms) to extract temporal patterns and dynamics extractions, and 6) image feature extraction/creation (e.g., flattened arrays with raw pixel intensities, color histograms, SIFT or HOG texture descriptors, deep learning feature embeddings such as Convolutional Neural Networks-extracted features) that convert image data into structured representations. For a more detailed overview of feature engineering approaches please consult Heaton (2016) and Verdonck et al. (2024).

On the other hand, feature selection, which is typically considered a subset of feature engineering, is the process of choosing a subset of relevant features from an existing set of features. It aims is to reduce dimensionality, avoid overfitting, improve interpretability, and speed up computation. Feature selection techniques include dimensionality reduction, which uses Principal Component Analysis (**PCA**) or forward/backward feature selection methods like Recursive Feature Elimination (**RFE**) to select features that alone or in combination improve the overall prediction performance of the models (Guyon and Elisseeff, 2003). Nevertheless, we must be aware that principal components enhance computational performance and reduce redundancy, but they obscure domain understanding and limit explanatory insights. Principal components can be powerful features in machine learning because they reduce dimensionality, mitigate multicollinearity, and often improve model efficiency and generalization by concentrating most of the data variance into a smaller set of uncorrelated variables. However, the trade-off is interpretability, since principal components are abstract linear combinations of the original features, making it difficult to relate model outputs back to meaningful, domain-specific variables. They are also data- and scale-dependent, so their definitions can shift across datasets, and they may fail to capture nonlinear structures in the data.

Discriminant analysis methods, such as Linear Discriminant Analysis (**LDA**), further support the feature engineering process by projecting data onto axes that maximize class separability, effectively highlighting the most relevant features. Clustering techniques, such as k-means or hierarchical clustering, can reveal structure in the data, guiding the creation of new features or the selection of representative variables. Feature engineering should be considered when a potential prediction quality gain or reduced overfitting can be secured. This typically occurs when the collected data includes: 1) complex variables that require splitting into simpler variables with more prediction power, 2) multiple variables that require aggregation to form new variables with enhanced prediction capabilities, or 3) a larger number of variables that requires subsetting to help reduce overfitting.

### Data normalization and transformation

Data normalization and transformation are critical preprocessing steps that enhance data consistency, reduce variability in input scales, and improve the performance of many machine learning algorithms, especially those sensitive to feature magnitudes, such as k-Nearest Neighbors, Support Vector Machines, shrinkage-based methods such as Ridge Regression, LASSO, or Elastic Net, and gradient descent-based models. Normalization ensures data consistency, reduces variable scale variations, and improves model performance, particularly for algorithms sensitive to scale differences among inputs. Common methods include min-max scaling that rescales data to values in the range [0,1] and Z-score standardization, which centers data around mean zero with unit variance (Cabello-Solorzano et al., 2023). In some cases, log transformations, Box-Cox (Box and Cox, 1964), or power transforms may also be applied to handle skewed distributions or reduce the influence of outliers. A careful descriptive statistics analysis of the data and solid knowledge of machine learning algorithm propensity and sensitivity to input scale differences are required to gain insights into whether normalization is necessary.

The resilience of machine learning regression models to abnormal data patterns, such as outliers, skewness, or scale discrepancies, varies significantly across algorithms. Models like Decision Tree **(DT)** and Random Forest **(RF)** tend to be more robust, based on data splitting rather than distance or gradient calculations. In contrast, models like linear regression **(LR)**, Support Vector Machine Regression (**SVM**), and Artificial Neural Networks (**ANN**) are more vulnerable to these irregularities, leading to inaccurate model coefficients, slow convergence, or unstable training. Although some models incorporate regularization techniques or robust loss functions to mitigate the effects of abnormal data, proper normalization and transformation remain essential to ensure accurate, reliable, and generalizable predictions. These preprocessing steps ultimately contribute to more interpretable outputs and improved model robustness in the presence of real-world data challenges.

## Fundamentals of Supervised Machine Learning Regression

### Overview of regression techniques

Regression is a fundamental technique in supervised machine learning that models the relationship between input variables (features) and one or more continuous output variables (targets). It is widely used in predictive modeling to estimate numerical outcomes based on historical data. Regression techniques can be broadly classified into linear and nonlinear methods. Linear regression assumes a linear relationship between input and target variables, making it computationally efficient and interpretable (Hastie et al., 2009). Nonlinear regression models, such as ANN and SVR, capture complex relationships that cannot be accurately represented using a straight line.

### Commonly used regression algorithms

In machine learning, regression algorithms are central to predictive modeling, each offering unique strengths depending on the nature of the data and the problem at hand. Among the most foundational is linear regression, which models the target variable as a linear function of input features. It serves as a useful baseline due to its simplicity and interpretability (James et al., 2013). However, simple or multiple linear regression assumes homoscedasticity, independence and linearity, which often do not hold in real-world data. When these assumptions are violated, extensions such as Weighted Least Squares (WLS) for heteroscedasticity and Generalized Least Squares (GLS) for correlated errors can be applied. Additionally, regularized regression methods such as Ridge (L2) and Lasso (L1) have been developed to address other limitations such as overfitting, generalization and multicollinearity, while they are not able to address violations of independence assumptions or non-linearity in the data. These methods add penalty terms to the loss function, shrinking coefficients to reduce overfitting and enhance model generalization (Tibshirani, 1996). Elastic Net, a compromise between Ridge and LASSO, is particularly effective when predictors are highly correlated (Zou and Hastie, 2005).

Beyond linear models, tree-based algorithms offer a powerful alternative for modeling complex, nonlinear relationships. Decision Tree regression builds hierarchical models by recursively partitioning the data, while Random Forest regression, an ensemble of decision trees, aggregates predictions from multiple trees to improve accuracy and robustness (Breiman, 2001). These models are effective in capturing nonlinearities and interactions among features and are also relatively interpretable compared to many other ML approaches because their structure closely mirrors human reasoning.

Further improvements to tree-based methods are Gradient Boosting Machines (**GBM**) and Extreme Gradient Boosting (**XGBoost**). These ensemble techniques build models sequentially, where each subsequent model attempts to correct the errors of the previous ones, using gradient descent to minimize the loss function (Chen and Guestrin, 2016). Their ability to model subtle patterns in data has made them highly popular in data science competitions and real-world applications alike.

The ANN represent another class of flexible and powerful regression tools, capable of modeling highly intricate and nonlinear relationships, particularly when trained on large datasets (McCulloch and Pitts, 1943; Goodfellow et al., 2016). Variants such as Convolutional Neural Networks (**CNN**) and Recurrent Neural Networks (**RNN**) further specialize in structured and temporal data, respectively. A more biologically grounded extension, Bayesian Regularized Artificial Neural Networks (**BRANN**), incorporates Bayesian inference into ANN training by assigning prior distributions to network weights. This approach helps regularize the model and yields more stable estimates, particularly valuable in applications like genome-wide association studies (Pérez-Rodríguez et al., 2013; Glória et al., 2016).

In addition to these, SVM regression offers a robust, margin-based learning framework that fits a function within a specified error margin using a kernel trick to handle nonlinearity (Smola and Schölkopf, 2004). Similarly, K-Nearest Neighbors (**KNN**) regression provides a simple, instance-based approach by predicting values based on the average of the nearest data points (Cover and Hart, 1967). While KNN is easy to implement and interpret, its performance is sensitive to the choice of the distance measure and the distribution of data. Gaussian Process regression (**GPR**), on the other hand, employs a Bayesian, non-parametric approach to model distributions over functions. GPR is particularly suitable for small datasets and offers valuable uncertainty quantification (Rasmussen and Williams, 2005).

Although each of these algorithms has unique advantages, such as interpretability, computational efficiency, or predictive power, they also come with limitations. No single method guarantees optimality across all contexts. Therefore, the process of selecting the most appropriate regression model is multifaceted. It requires thoughtful data preprocessing, including cleaning and sampling, rigorous model tuning and evaluation, and robust validation strategies. Ultimately, successful predictive modeling hinges on a deep understanding of both the data and the algorithmic tools available.

### Evaluation measures for regression models

The evaluation of predictive performance for regression models is assessed using various performance measures, which are often mistakenly referred to as “metrics,” despite most not meeting the strict mathematical definition of a metric. Strictly speaking, a function d(x,y), where x and y are two inputs, qualifies as a *metric* only if it satisfies three mathematical conditions: 1) *non-negativity*: d(x,y)≥0, with equality if and only if x=y, 2) *symmetry*: d(x,y)=d(y,x), and 3) *triangle inequalit*y: d(x,y)≤d(x,z)+d(z,y), for any third point z. However, most commonly used performance measures in machine learning do not satisfy all of these conditions. For instance, the Pearson correlation coefficient violates the non-negativity condition as it can take negative values, while standard regression measures such as mean absolute error (**MAE**) and root mean squared error (**RMSE**) typically fail the triangle inequality. Similarly, classification accuracy, often mislabelled as a metric, also does not meet this criterion. Therefore, while these measures serve as practical tools for evaluating model performance, they should not be mistaken for true mathematical metrics.

Regression model evaluation measures can generally be classified into three categories based on their purpose: error-based measures, correlation-based measures and explanatory coefficients. For a more detailed overview of evaluation measures used in mathematical modeling within livestock science, refer to Tedeschi (2006). Common error-based measures include the MAE, which measures the average absolute difference between actual and predicted values, the mean squared error (**MSE**) and RMSE, which penalize larger errors more heavily than MAE, and the mean absolute percentage error (**MAPE**), which expresses errors as a percentage of actual values and it particularly useful for enhanced interpretability.

Common correlation-based measures include the Pearson product-moment correlation coefficient (**PCC** or ρ), which measures the linear correlation between the raw values of two variables (Pearson, 1895). The coefficient represents the ratio between the covariance of two variables and the product of their standard deviations and has values between −1 (strong inverse correlation) and 1 (strong direct correlation), while a value of 0 represents no correlation. Another popular correlation measure is Lin’s concordance correlation coefficient (**CCC**), which measures the agreement between the raw values of two variables. CCC is equivalent to 1 minus the ratio of the expected orthogonal squared distance from the 45-degree line (*y* = *x*) and the expected orthogonal squared distance from the 45° line (*y* = *x*), assuming independence (Lin, 1989). This allows CCC to capture not only the correlation between the two variables but also the slope of the general direction of the correlated values, thus exceeding the capabilities of the PCC. CCC has values between −1, which indicates strong discordance and 1, which represents perfect concordance, while a value of 0 indicates no concordance.

Common explanatory coefficients include *R*^2^ and adjusted *R*^2^, which indicate the proportion of variance explained by the model (James et al., 2013). In standard linear regression, R-squared represents the proportion of the variation in the response (dependent on inputs) variable that is predictable from the explanatory (typically independent of each other) variables and typically takes values between 0 (independent variables do not explain the predicted dependent variable) and 1 (independent variables explain perfectly the predicted dependent variable). Occasionally, a regression model can produce values of *R*^2^ outside the range 0 to 1, which typically signals that the model fits the data worse than the worst possible least squares predictor. A negative *R*^2^ indicates that the model performs worse than a baseline model that predicts the mean of the response variable for all observations, and thus, explains less variance than predicting the mean, indicating a poor fit. This situation arises when the residual sum of squares exceeds the total sum of squares, leading to an *R*^2^ value less than zero. This typically indicates a poor model choice or other model or data-related issues during the modeling steps. However, when applied to non-linear models or mixed-effects models with both fixed and random effects, the interpretation of *R*^2^ is less straightforward. In these cases, different formulations of *R*^2^ such as marginal *R*^2^ for fixed effects and conditional *R*^2^ for both fixed and random effects have been proposed, and values may not carry the same intuitive meaning as in simple linear regression (Nakagawa and Schielzeth, 2013). Thus, while *R*^2^ remains a useful summary statistic, its interpretation depends on the modeling framework and should be applied with caution outside standard linear regression.

The adjusted *R*^2^ measures are typically employed to address the inflation of *R*^2^ scores caused by an increase in input variables. Therefore, adjusted *R*^2^ is highly recommended to be used when performing comparisons among regressor predictions produced by models with different numbers of input variables.

## Model Evaluation and Pipeline Construction for Regression Modelling

### Defining the pipeline workflow

**Figure 1.**
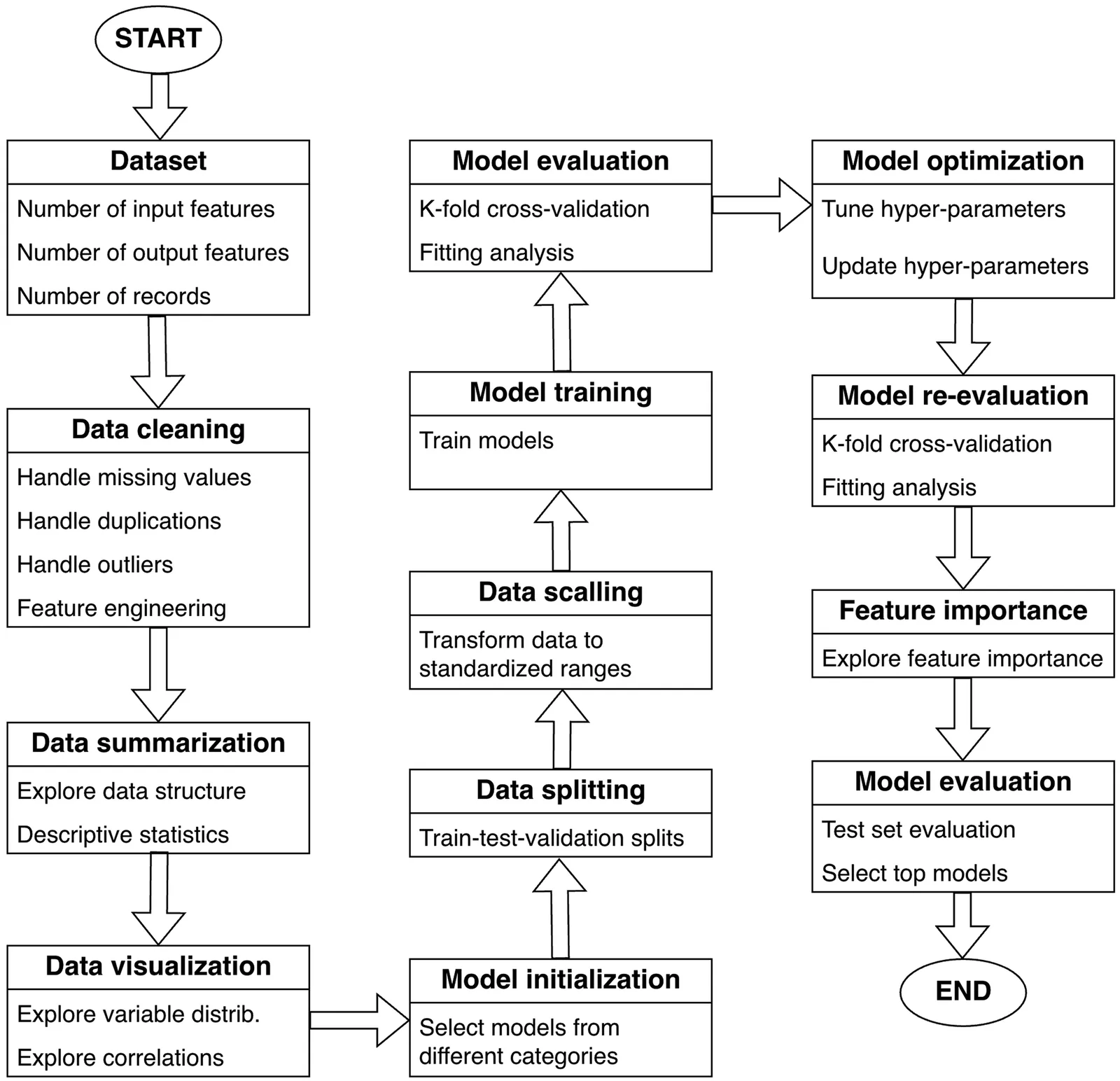
Machine learning regression pipeline. The pipeline includes some of the most prominent steps required to be completed when modelling a dataset with the aid of machine learning.

A regression pipeline integrates data preprocessing, feature selection, model training, model optimization and evaluation steps into a streamlined process that requires careful design and consideration (Kuhn and Johnson, 2013). A generalized regression pipeline that includes all these steps is presented in Figure 1, and supporting Python code for implementing this computational pipeline is provided in Supplemental Materials.

### Data splitting strategies (train-test, cross-validation)

Constructing modeling pipelines and model evaluation for predictive purposes requires careful consideration of data splitting strategies, particularly when datasets have complex structures. The ML modeling often considers two common complementary approaches supporting model evaluation and construction: train-test split and *k*-fold cross-validation (CV).

An initial train-test split approach is performed, dividing the dataset into training and test sets. While there is no general rule on what is the optimal ratio that should be used for the split (typically 80–20%), the decision is typically made based on the size of the dataset, where larger training set percentages are recommended for smaller datasets to increase the model performance. Due to inherent variability in data splits caused by data distributions, the train-test split process is recommended to be performed more than once, and the average and standard deviation of the final results must be reported. Alternatively, resampling methods, such as bootstrapping or jackknifing techniques can also be used.

Once a training set is defined, the model construction process can begin. A *k*-fold cross-validation process is employed, representing a robust method to evaluate model performance by dividing data into k subsets, training *k*-1 subsets, and validating them on the remaining subset (James et al., 2013). The process is repeated *k* times, and the average and standard deviation of the results are reported and analyzed to evaluate the model performance. A robust model will have a prediction performance characterized by a high average and low standard deviation. The number of folds, *k*, will decide the size of the data subset used for model construction and the number of models that need to be built to properly validate their performance. Choosing the number of folds (*k*) is typically made based on the overall size of the dataset (lower *k* for larger datasets), the availability of computing power (lower *k* for limited computing power), and the time limitations inherent to all projects (lower *k* for tighter project deadlines).

Importantly, when datasets exhibit complex hierarchical or clustered structures, such as multiple farms, animals nested within farms, repeated measurements per animal, different breeds, or varying treatments, standard random splitting may lead to information leakage and overly optimistic performance estimates. In such cases, the data splitting strategy must account for the dependencies in the data. Such strategies include:

- Grouped cross-validation, which ensures that all observations from the same group (e.g., the same animal, farm, or breed) appear entirely in either the training or test set. This prevents the model from “seeing” correlated observations in both training and validation, which could artificially inflate predictive performance.
- Repeated measures/nested CV: For longitudinal data with repeated measurements per animal, nested CV or leave-one-animal-out strategies can be applied to respect within-animal correlation.
- Stratified sampling: When outcomes are unbalanced across treatments, breeds, or farms, stratification ensures that each fold reflects the overall distribution of these factors.

Several studies in animal science and dairy research have emphasized the importance of considering hierarchical structures in CV design (Coelho Ribeiro et al., 2021; Yilmaz Adkinson et al., 2024; Wang et al., 2025), highlighting that ignoring these dependencies can bias performance measures and lead to misleading conclusions. Implementing structure-aware CV strategies improves the robustness and generalizability of predictive models in real-world, multilevel datasets.

In summary, while traditional train-test splits and *k*-fold CV remain fundamental tools, careful consideration of the data structure, clustering, and repeated measures is essential for valid performance estimation. Adopting grouped, stratified, or nested CV strategies ensures that evaluation measures accurately reflect the predictive ability of models on independent data, particularly in the context of complex biological or agricultural datasets.

### Model selection and hyperparameter tuning

In ML modeling, parameters are the values the model learns during training, such as weights and biases in neural networks, the feature entropy and information gain in decision trees, the complexity parameter in regularized regression and the decay parameter in kernel regression. These values are automatically updated during training to minimize a loss function. Hyperparameters are external settings data scientists set before training, influencing the model’s structure, learning process, and performance. Examples include the learning rate and the number of layers in a neural network or the maximum depth of a decision tree. Selecting the best model involves hyperparameter optimization techniques like *Grid Search*, which exhaustively searches for the best combination of values for the hyperparameters of a model, *Random Search*, which performs a randomized search of various hyperparameter combinations and *Bayesian Optimization*, which applies a probabilistic approach to find optimal hyperparameter settings (Snoek et al., 2012). For a larger selection of optimization algorithms including simulated annealing, genetic algorithms and tabu search we recommend the reader to consult the work of Hoos and Stuetzle (2005).

Hyperparameter tuning is often applied with *k*-fold cross-validation (a process known as double or nested cross-validation) to avoid optimistically biased model performance evaluations caused by using the same cross-validation procedure and dataset to both tune and select a model. Nevertheless, while the use of nested cross-validation could be beneficial and increase the generalizability while avoiding over-fitting of your models (Cawley and Talbot, 2010), it comes at a high computational cost, requires significantly larger datasets and sometimes shows limited benefits and its necessity should be seriously considered for practical scenarios (Wainer and Cawley, 2018).

### Model fitting analysis

The analysis of the ability of a model to fit the data can be achieved using various techniques such as learning curves (Webb et al., 2011). Verifying the fitting quality of machine learning predictive models is highly recommended and necessary regardless of the theoretical guarantees associated with some prediction algorithms (Viering and Loog, 2023).

**Figure 2.**
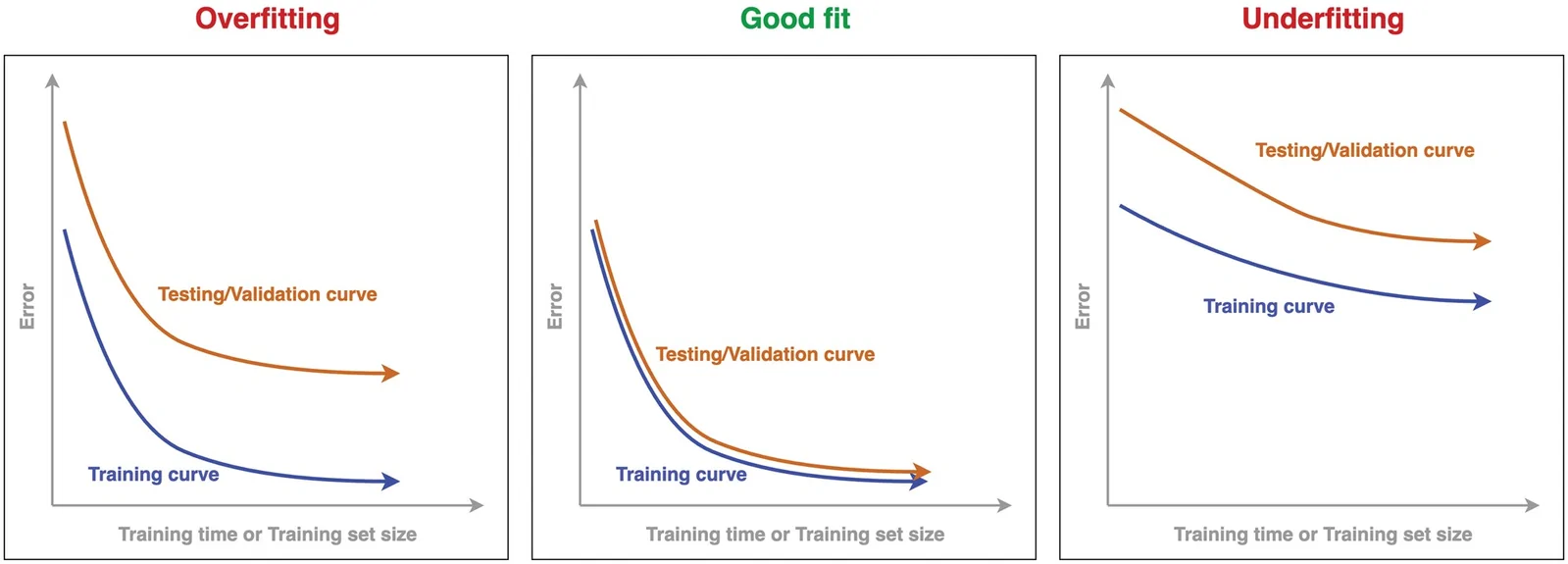
Learning curves depicting model overfitting (left), good fit (center) and underfitting (right). Testing/validation curves are depicted with orange while training curves are colored in blue.

A learning curve represents the performance of a model over a predefined and incremental number of iterations or as a function of the training set size, and it is used to diagnose issues such as underfitting or overfitting. A graphical representation of learning curves, as shown in Figure 2, typically includes a training learning curve depicting the model performance during model training and a validation or a testing learning curve showing the model performance on an unseen dataset. The presence of a significantly large gap between the training and the validation/testing curves signals overfitting where the training error is typically low and the validation/testing error is high (Figure 2—left plot). From the perspective of the bias-variance trade-off, this indicates high variance: the model is overly complex relative to the data and captures noise along with the signal, leading to poor generalization. In contrast, high training and validation errors indicate underfitting (Figure 2—right plot) since the model is too simple to capture the patterns in the data. This scenario corresponds to high bias, where the model assumptions prevent it from representing the true underlying relationships. If the two curves converge at a low error level, this indicates a good fit (Figure 2—center plot).

The shape of the learning curves (decreasing or increasing from low to high set sizes or training times) depends on the measure used for model evaluation typically represented on the y-axis. For instance, if a measure like *R*^2^ is plotted, which increases with model performance, learning curves will show an upward trend with increasing training size or time. Moreover, learning curves may sometimes appear jagged or irregular. This variability can result from factors such as high data variance, an inappropriate learning rate, small batch sizes, or poorly representative training and validation datasets. Here, we recommend practitioners to investigate the fitting of the models before and after optimization steps to carefully direct the optimization process towards the models that require it the most while gauging and reporting the benefit of the optimization concerning performance and over- and under-fitting.

### Model feature importance, interpretability and explainability

Feature importance in machine learning refers to techniques that assign scores to input features based on their contribution to a model’s predictive performance. They often help with model explainability by providing insights into the features that influence model predictions (Ribeiro et al., 2016; Lundberg and Lee, 2017). Feature importance also helps with understanding the model, improving its performance, and reducing the dimensionality of the dataset.

There are three main categories of feature importance techniques, and they differ depending on the type of model used in the study: 1) model-specific, 2) model-agnostic, and 3) regularization-based.

Models-specific feature importance techniques work only with certain model types such as tree-based models (e.g., DT, RF, XGBoost) and linear models (LR). In regression decision tree models, the reduction in variance at each split measures how much each numerical feature decreases the prediction error, providing an indication of the importance of that particular feature. In the XGBoost algorithm, the performance gain representing the improvement in accuracy brought by a feature to the branches it is on also reflects feature importance. In linear models, feature importance can be assessed using the magnitude of the coefficients, typically after data normalization. However, one may also consider the coefficients relative to their standard errors (t-statistics) or their *P*-values to account for uncertainty and statistical significance, rather than relying solely on their absolute or nominal values.

Model-agnostic techniques have a larger utility span and can be applied to any ML model. For example, one of the most popular approaches, Permutation Feature Importance (**PFI**) is applied to identify the features most relevant to the output for each model. Each feature is randomly shuffled while keeping all the other features unchanged, and the increase in prediction error is measured. Features that cause a larger increase in error are considered more important to the model. Another popular technique based on cooperative game theory, SHapley Additive exPlanations (SHAP), assigns each feature an importance value based on its contribution to a specific prediction. The technique uses concepts from game theory—specifically Shapley values—to fairly distribute the prediction outcome among all features by averaging their marginal contributions across all possible combinations. SHAP is model-agnostic (with specialized versions for tree, linear, and deep models), provides both local and global interpretability, and produces intuitive visualizations, making it one of the most robust and consistent tools for understanding model behavior.

Embedded and regularization-based feature selection integrate the selection process directly into model training, allowing the algorithm to automatically identify and weigh important features. These methods often rely on regularization techniques, such as L1 regularization in the least absolute shrinkage and selection operator (**LASSO**) and L2 regularization in Ridge Regression (Tibshirani, 1996), which add a penalty to the model’s loss function to constrain the magnitude of the coefficients and reduce overfitting. L1 regularization tends to produce sparse models by driving some coefficients exactly to zero, effectively performing feature selection, whereas L2 regularization shrinks all coefficients toward zero without eliminating any, which stabilizes estimates but retains all features. This not only improves model generalization but also reduces complexity. Embedded methods are efficient and model-specific, commonly used in linear models, decision trees, and some ensemble algorithms such as Light Gradient-Boosting Machine (LightGBM), where feature importance is inherently evaluated during the learning process.

The use of agnostic models is highly recommended due to their increased versatility and reduced model selection limitations; nevertheless, other techniques can be also considered when applicable. The case studies below are intended to provide concrete examples of how generic regression ML pipelines can be applied to different livestock-related datasets to build and evaluate effective predictive models.

## Case Studies and Applications in Livestock Research

In the following sections, we present examples to demonstrate how open-source Python code can be used to streamline data processing steps, statistically analyze data, train, validate, and test predictive models using machine learning algorithms applied to body weight prediction in pigs and dry matter intake prediction in hair sheep.

To investigate predictive modeling based on morphometric or other numerical data, we developed a machine learning pipeline tailored for regression problems where only a single measurement per animal is available. The pipeline was implemented in Python (version 3.11.9) using *Scikit-learn* version 1.5 (Pedregosa et al., 2011) and additional scientific and graphic libraries such as *scipy (version 1.13.1)*, *statsmodels (version 0.14.2)*, *numpy (version 1.26.4)*, *pandas (version 2.2.2)*, *matplotlib* and *seaborn*, and it automates model training, optimization, evaluation, and comparison across four regression algorithms. The pipeline can be easily extended to include more algorithms and evaluation measures and it is made publicly available in the Supplemental Materials.

***Data preparation:*** The script performs basic quality control steps such as identifying and removing duplicated rows and columns, and encoding categorical input variables. It assumes that significant data cleaning is conducted prior to execution. Input datasets must contain only numerical values and be free of missing entries or formatting inconsistencies. For simplicity, convenience and easiness to use our code, the response variable (ie, the target for regression) is expected to be located in the last column of the dataset. Data is read from a CSV-formated file using the Python *pandas* library. After reading the dataset, the pipeline performs a series of preprocessing operations:

- *Train-test split*: The data is randomly split into training and test subsets (default: 80/20), ensuring reproducibility with a fixed seed (random_state = 1).
- *Feature scaling and encoding*: Standardization is applied to all predictor variables. Categorical variables are encoded into consecutive integer numerical values using the sklearn LabelEncoder function. While this approach is computationally efficient and ensures numerical values will replace categorical ones in the dataset, it is not without limitations. For example, it is not recommended for features with nominal values since it can imply a false order and lead to results misinterpretations. Instead, the one-hot encoding should be used where each unique category is represented by a binary column with a value of 1 indicating its presence and 0 indicating its absence. By default, StandardScaler is used to center the data and scale to unit variance, which improves the convergence and performance of many regression algorithms. Alternatives such as MinMaxScaler and RobustScaler are included in the code but commented out, allowing flexibility depending on the presence of outliers.
- *Data shuffling*: Shuffling is enabled during splitting to avoid any inherent ordering bias in the dataset.

These preprocessing steps ensure the dataset is normalized and well-prepared for model training, reducing bias and improving generalization performance.

***Model training and evaluation:*** The pipeline splits the dataset into training and testing sets and standardizes the features using one of several available scalers (*StandardScaler*, *MinMaxScaler*, *RobustScaler*). It supports several regression algorithms, including linear regression, DT regressor, KNN, and SVR. Each model is trained using either cross-validation or a combination of random/grid search and repeated *k*-fold validation to optimize hyperparameters. Performance evaluation measures include MAE, MSE, RMSE, MAPE, and *R*^2^. Additionally, the script calculates CCC for assessing model agreement.

***Model interpretability and saving*** *:* The pipeline includes tools for visualizing learning curves and variable importance via permutation feature importance. It also generates quantile-quantile (Q-Q) plots for assessing normality of residuals. Unlike classical statistical models, where the assumption of normally distributed residuals underpins valid inference and hypothesis testing, most machine learning models do not rely on residual normality. It is not necessary to check residual normality in ML for predictive tasks, since predictive algorithms don’t rely on it. However, while assessing residuals in ML is not required for predictive validity, residual analysis remains useful for understanding model limitations, error patterns, and overlooked data structures, especially in applied fields like animal science where prediction errors may carry practical implications. For example, systematic patterns in residuals may indicate non-linearity, omitted features, or unmodeled interactions, while a changing residual spread can reveal heteroscedasticity. Residual autocorrelation may signal temporal or spatial dependence, and large deviations can highlight outliers or influential points. Moreover, clustered or stratified residual patterns may uncover hierarchical structures (e.g., farms, herds, breeds) or repeated measures effects that the model failed to capture. Trained models and associated scalers are serialized using the Python *joblib (version 1.4.2)* library for future use or deployment.

### Case study 1: Swine body weight prediction

Live body weight (LBW) is a key indicator used to estimate growth, feed conversion efficiency, body condition, and disease presence and to guide decisions related to housing, nutrition, and health management across different stages of livestock development. Estimating swine body weight using body measurements is valuable because it provides a low-cost and accessible alternative to weighing scales, which can be expensive or impractical in smallholder or remote settings. Body measurements are typically taken manually with a measuring tape (e.g., heart girth, body length, or leg length), requiring minimal equipment and setup compared to moving animals onto a scale. Although this method still involves some handling, it can be less stressful than weighing, as animals do not need to be restrained or forced onto a platform. In addition, it enables more frequent or large-scale monitoring when scales are unavailable. It also empowers farmers to make informed decisions about feeding, medication, and marketing and provides valuable data for research, genetic improvement, and herd management.

This case study uses data from Marshall et al. (2023), collected from 765 pigs of various ages, sexes, and breeds across 157 smallholder households in five Ugandan districts to capture regional variation in pig characteristics. Within each district, administrative units with high pig populations and no active African Swine Fever outbreaks were identified, and households with at least two pigs were randomly selected for participation. All eligible pigs were measured, excluding visibly pregnant, aggressive, sick, or injured animals, with a limit of three randomly chosen piglets per litter. Data collection took place between November 29, 2021, and January 5, 2022, involving adult household members, 56% of whom were female. Pig live weight was recorded using a digital scale, while body measurements—including heart girth, height, and length—were taken in centimeters using tape and measuring sticks. Additional data included pig characteristics (sex, age, breed type, castration/pregnancy status, parity, housing type, and body condition score), as well as farmer demographics and measured pig weight. Breed was categorized as “local” or “exotic” based on enumerator observation. Due to data sparsity in some variables, this study focuses on five input features—age, heart girth, height, length, and body condition score—and uses live weight as the output variable. After removing one record with a zero-length measurement, the final dataset consists of 764 pigs. The goal of this case study is to process the raw data, train and evaluate machine learning models, and predict live body weight.

***Data preparation:*** During the data preparation phase, raw files are imported into Python, where extraneous header information is removed, and any duplicate records or columns are eliminated. Outliers are identified and excluded for all numerical variables using the Z-score method, which filters out records that fall more than four standard deviations from the mean. The resulting cleaned dataset, containing 752 records, is saved to a new file for use in subsequent analyses.

**Figure 3.**
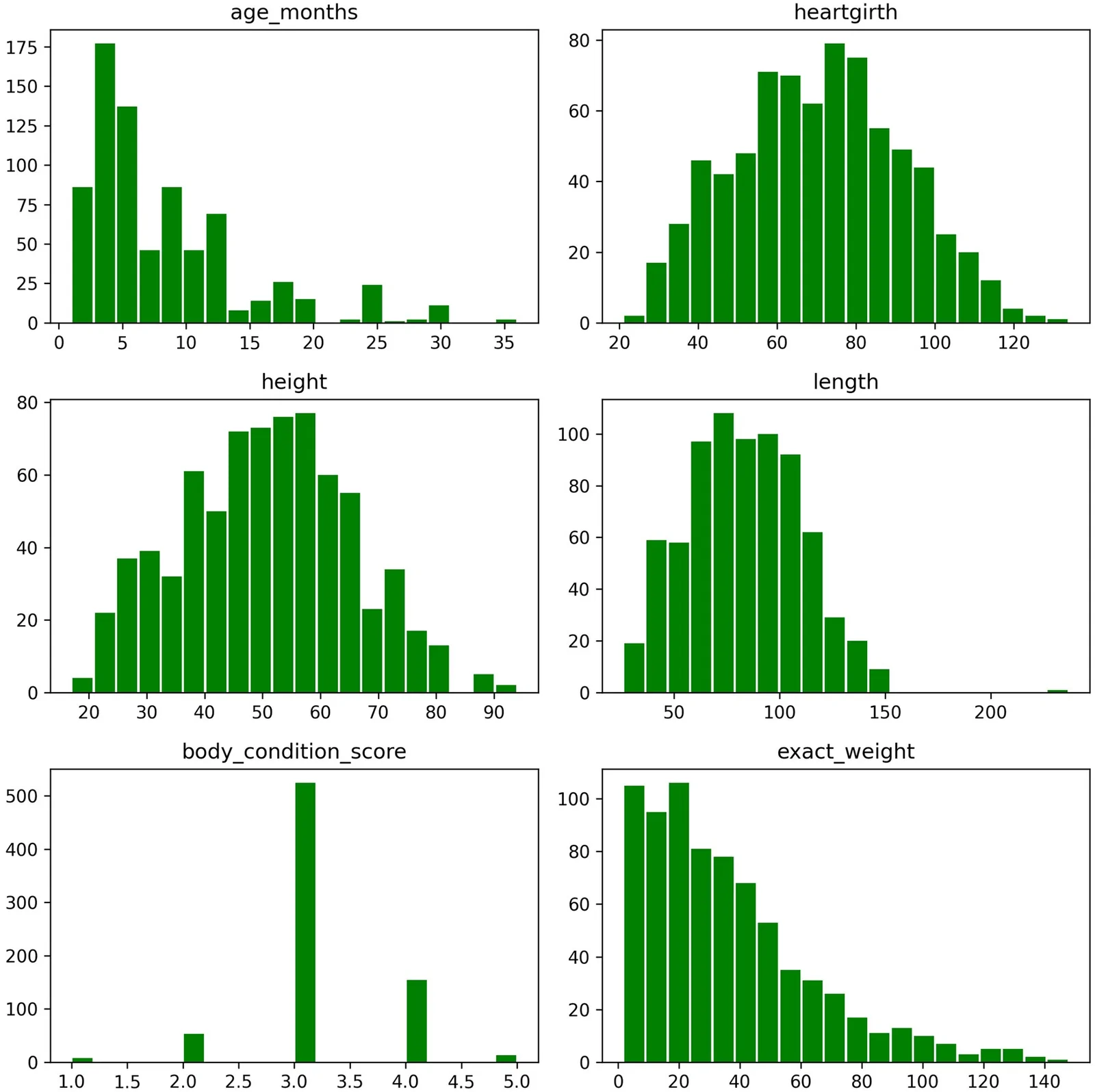
Case study 1—Variable distributions. The histograms represent the distribution of age (months), heart girth, height, length, body condition score and body weight (kg) collected from 752 pigs in a study by Marshall et al. (2023).

**Figure 4.**
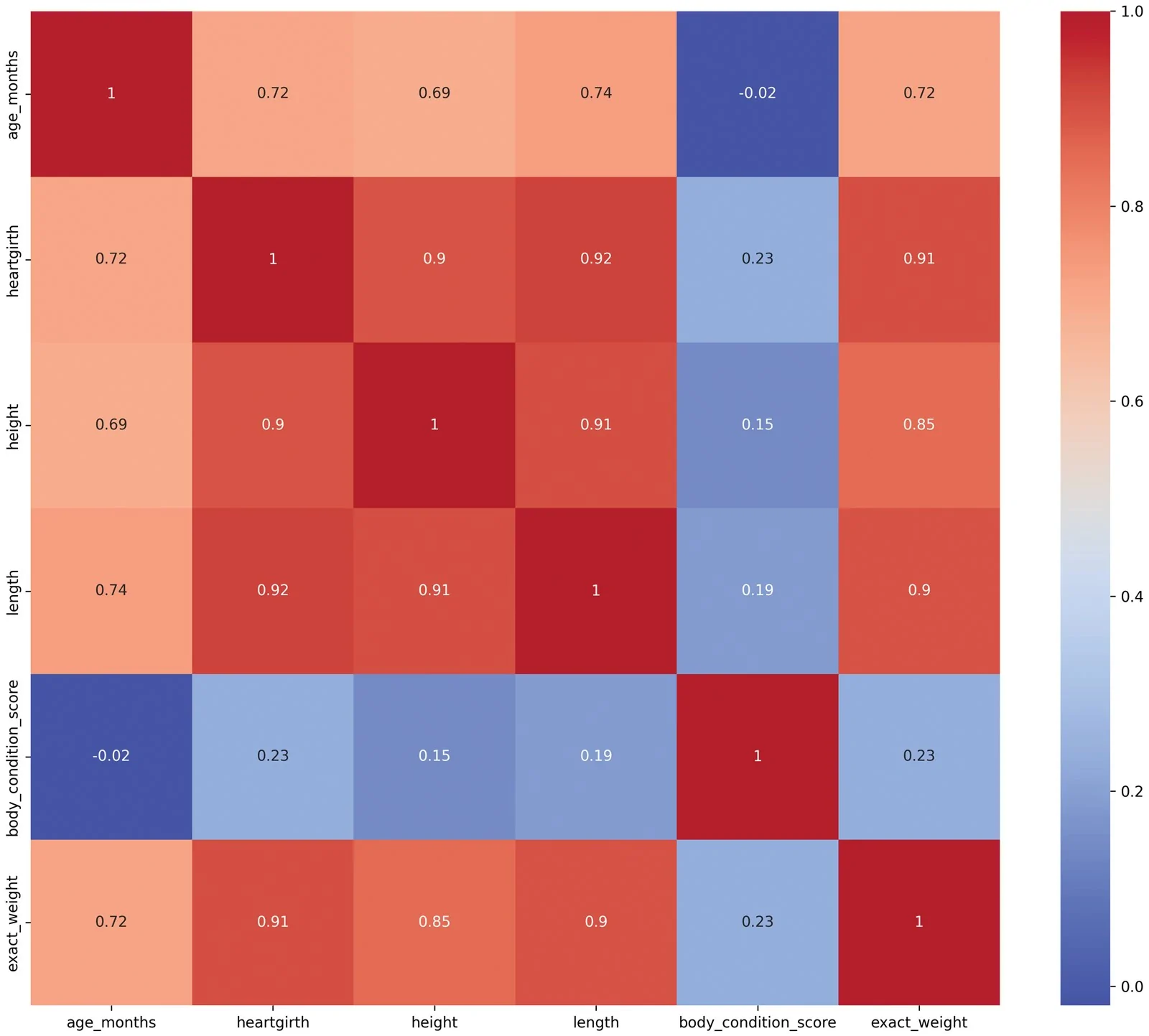
Case study 1—Pearson product-moment correlation scores for pairs of variables. The figure depicts Pearson correlation scores among all pairs of variables representing the age (months), heart girth, height, length, body condition score and the body weight of 752 pigs used in a study by Marshall et al. (2023).

***Data visualization*** *:* Distributions of individual variables are visualized (Figure 3), and Pearson product-moment correlation coefficients are computed for all variable pairs, with the results presented in a correlation plot (Figure 4). Heart girth, body length, and height show a strong correlation (0.85–0.91) with body weight, while age demonstrates a moderate correlation (0.72), and body condition score (BCS) shows a weak association (0.23) with the outcome variable.

***Model development and evaluation:*** The training dataset comprises 601 records (80% of the total), using five input variables—age (in months), heart girth, height, and length (in centimeters), and body condition score (on a scale of 1 to 5)—to predict the output variable, body weight (in kilograms). Five commonly used machine learning algorithms (KNN, LR, DT, RF, and SVM) are applied. Model performance is evaluated using repeated 5-fold cross-validation (3 repeats) and a separate test set containing the remaining 20% of the data (151 records). In this example, model performance is assessed using MAE, though other evaluation measures may also be considered.

***Hyperparameter optimization (HPO):*** Each machine learning model includes user-defined parameters, known as hyperparameters, which can be tuned to improve performance. In this example, a grid search approach evaluating all possible parameter combinations is used to identify the optimal settings. A complete list of the hyperparameter values tested and their best-performing values is provided in Table 1.

**Table 1.** Case study 1—List of hyperparameters, ranges of values and best settings for five ML algorithms

Algorithm hyperparameters	Range of values	Best value
*Linear Regression*		
**fit_intercept**	[True, False]	True
*K-Nearest Neighbour*		
**n_neighbors**	[1 : 10]—integers	10
*Decision Tree*		
**criterion**	[‘friedman_mse’, ‘absolute_error’, ‘poisson’, ‘squared_error’]	‘absolute_error’
**max_depth**	[1 : 9]—integers	5
*Support Vector Machine*		
**C**	[0 : 1.6]—increments of 0.2	1.4
*Random Forest*		
**n_estimators**	[20, 50, 100, 150, 200]	200
**max_depth**	[2 : 10]—integers	5

The best values for each hyperparameters were obtained using a grid search approach where all possible hyperparameter value combinations are attempted for each algorithm. The mean absolute error was used in this optimization process.

**Figure 5.**
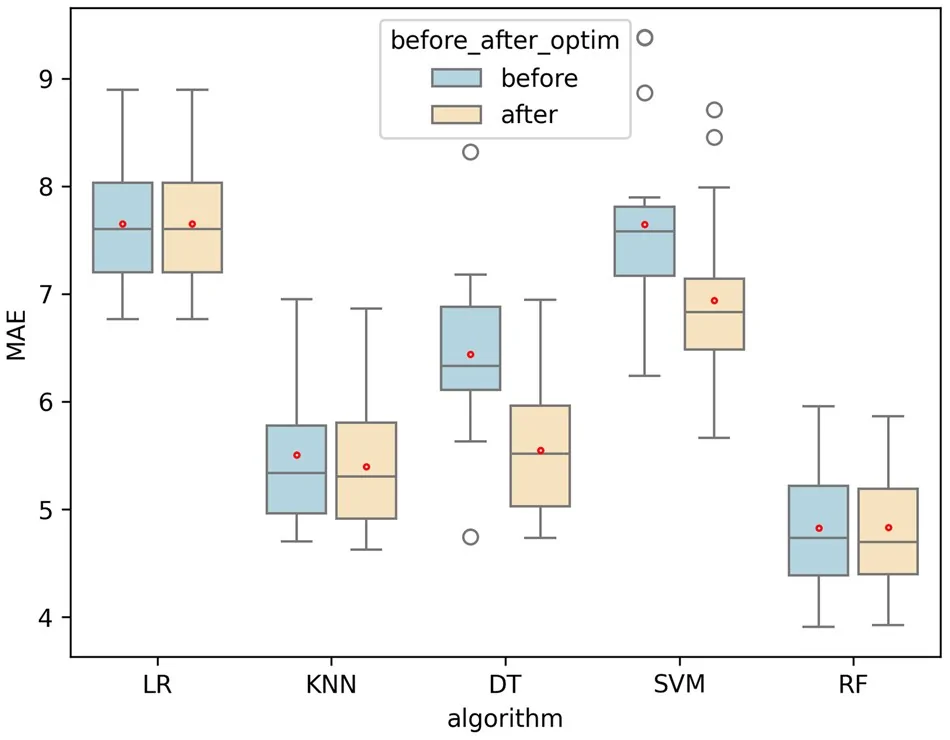
Case study 1—Cross-validation algorithm performance before and after hyperparameter optimization. The figure depicts box plots representing the MAE results for BW predictions obtained with five ML algorithms (Linear Regression, K-Nearest Neighbour, Decision Tree, Support Vector Machine and Random Forest) applied on a dataset published by Marshall et al. (2023).

**Figure 6.**
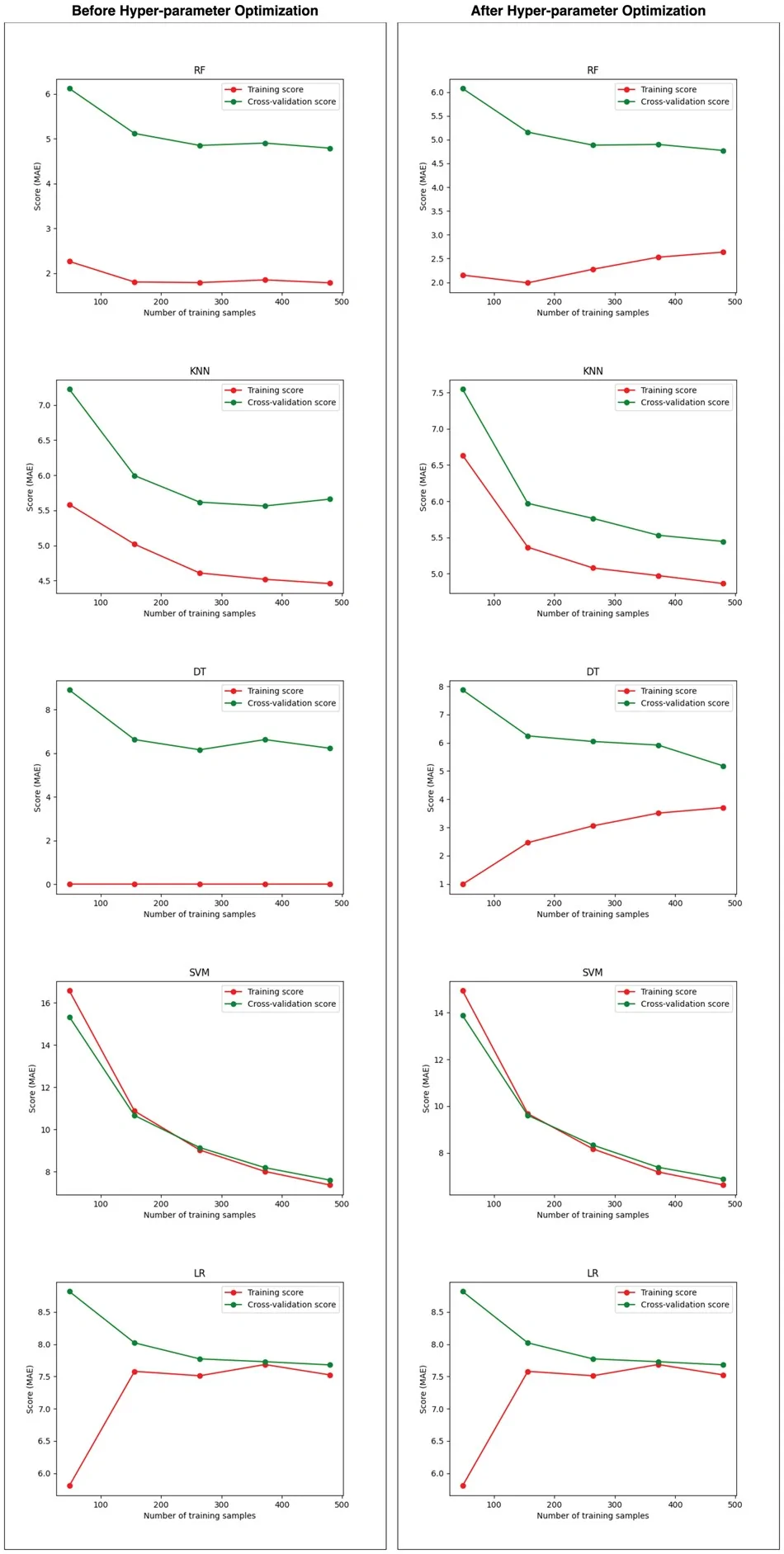
Case study 1—Learning curves depicting fitting performance for five machine learning algorithms predicting BW. The five machine learning models are Linear Regression, K-Nearest Neighbour, Decision Tree, Support Vector Machine and Random Forest.

***Prediction results and fitting analysis*** *:* Among the five algorithms tested, Random Forest achieved the best performance, with the lowest MAE of approximately 4.80 kg during cross-validation (both before and after hyperparameter optimization), and 4.68 kg on the test set. It was followed by k-nearest neighbors and decision tree, while support vector machine and linear regression performed the worst, both with MAE values exceeding 6.72 kg. Detailed results are presented in Table 2 and Figure 5. To evaluate model generalization, learning curves were used (Figure 6), revealing varying degrees of overfitting among the top-performing models, especially prior to hyperparameter optimization. Overfitting levels were visually assessed based on the gap between training and validation curves using MAE-based thresholds: No (0–2 MAE), Low (2–4 MAE), Medium (4–6 MAE), and High (>6 MAE). In contrast, Support Vector Regression and Linear Regression showed minimal signs of overfitting. Hyperparameter tuning significantly improved the performance and generalization of the top four models.

**Table 2.** Case study 1—Algorithms cross-validation and testing performance

Algorithm	Cross-validation MAE (std) before HPO	Overfitting	Cross-validation MAE (std) after HPO	Overfitting	Testing MAE after HPO
**Random Forest**	4.81 (0.48)	High	4.80 (0.56)	Medium	4.68
**K-Nearest Neighbour**	5.51 (0.65)	Medium	5.40 (0.59)	Low	4.63
**Decision Tree**	6.39 (0.72)	High	5.47 (0.69)	Low	5.17
**Support Vector Machine**	7.65 (0.91)	No	6.94 (0.84)	No	6.72
**Linear Regression**	7.65 (0.64)	No	7.65 (0.64)	No	7.27

For each of the 5 algorithms we report the validation MAE (kg) and overfitting results before and after hyperparameter optimization and the testing MAE results. HPO, hyperparameter optimization; MAE, means absolute error; std, standard deviation.

**Figure 7.**
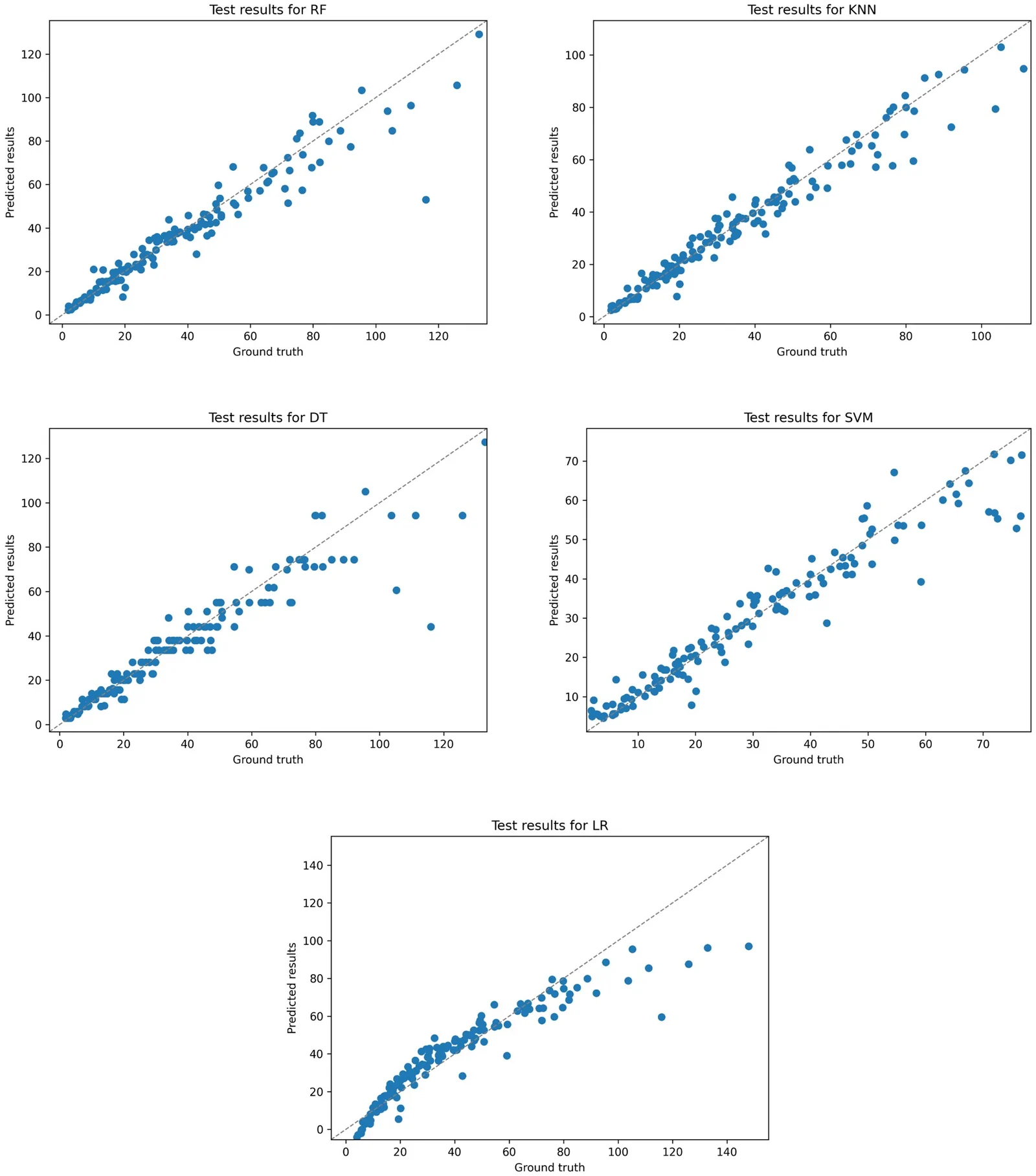
Case study 1—Scatter plots representing the predictions of five machine learning algorithms on the testing set. The five machine learning models are Linear Regression, K-Nearest Neighbour, Decision Tree, Support Vector Machine and Random Forest. Each plot represents predicted versus ground truth body weights (measured in kilograms) of 151 pigs from a study by Marshall et al. (2023).

**Figure 8.**
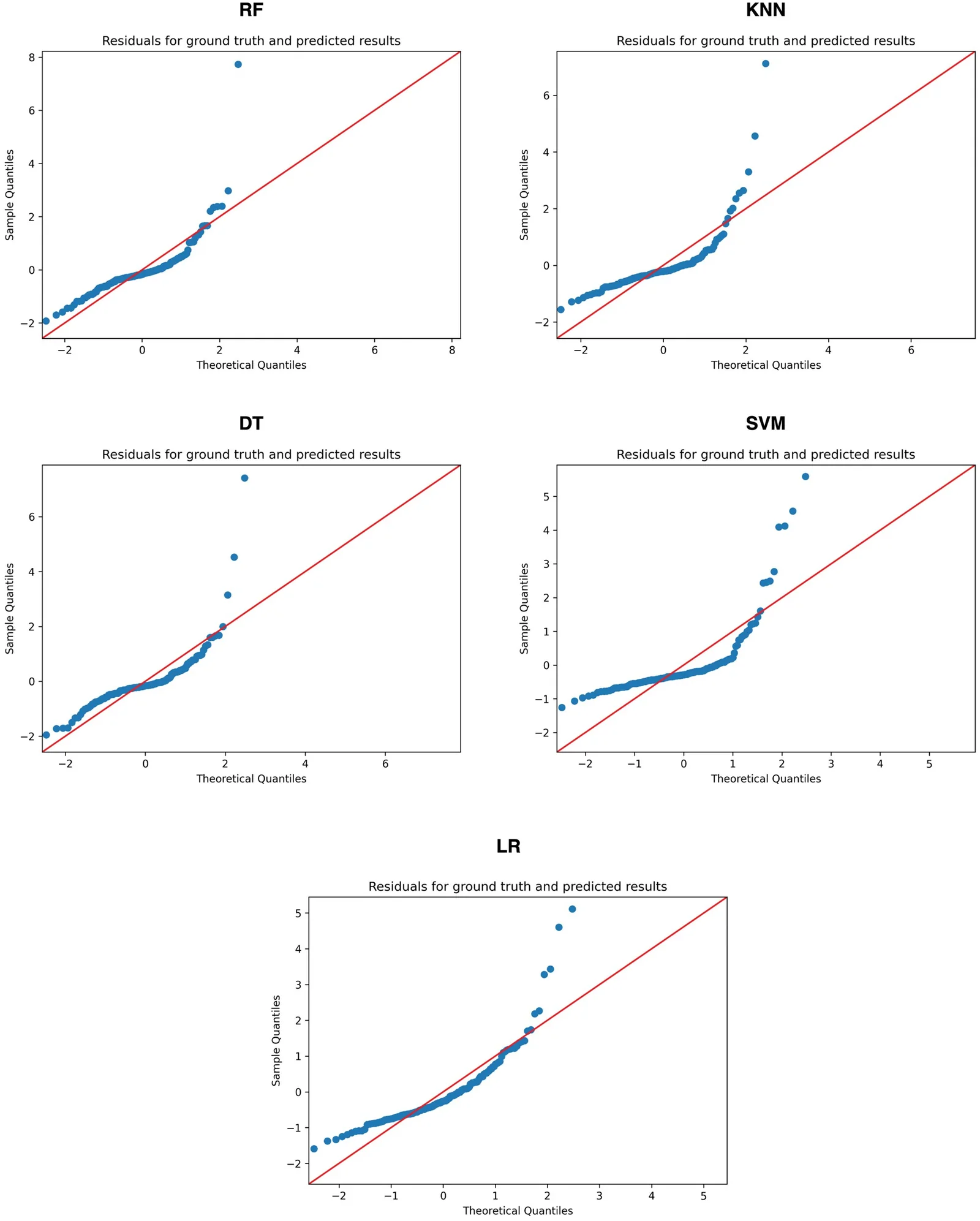
Case study 1—Quantile-Quantile plots for errors resulting from the five machine learning models applied on the testing set. The five machine learning models are Linear Regression, K-Nearest Neighbour, Decision Tree, Support Vector Machine and Random Forest. In each plot, the red line represents f(x) = x, which indicates where points would fall if the data followed the theoretical distribution (t-distribution) exactly. The blue dots represent the body weight residual values. The body weights were predicted based on a test set including 151 pigs from a study by Marshall et al. (2023).

Scatter plots and quantile-quantile (Q–Q) plots were generated to further explore prediction accuracy and error behavior (Figures 7 and 8). All five models performed well on small to medium-sized pigs but consistently under-predicted the weight of larger pigs. The Q-Q plots confirmed deviations from the expected normal distribution of errors, suggesting the models struggle to generalize across the full range of body weights, particularly at the higher end, for which fewer datapoints were available (Figure 3—bottom right).

These findings emphasize the importance of conducting fit diagnostics, even for well-established algorithms with strong theoretical foundations. However, this case study does not include a formal robustness analysis of the predictions on test data. Such analysis can be performed by repeating the modeling process with multiple randomized train-test splits (while keeping the same proportions) and averaging the results, though this comes at the cost of reduced reproducibility. To enable this, one can simply remove the *random_state = 1* setting from the *train_test_split()* function in the Python script.

**Figure 9.**
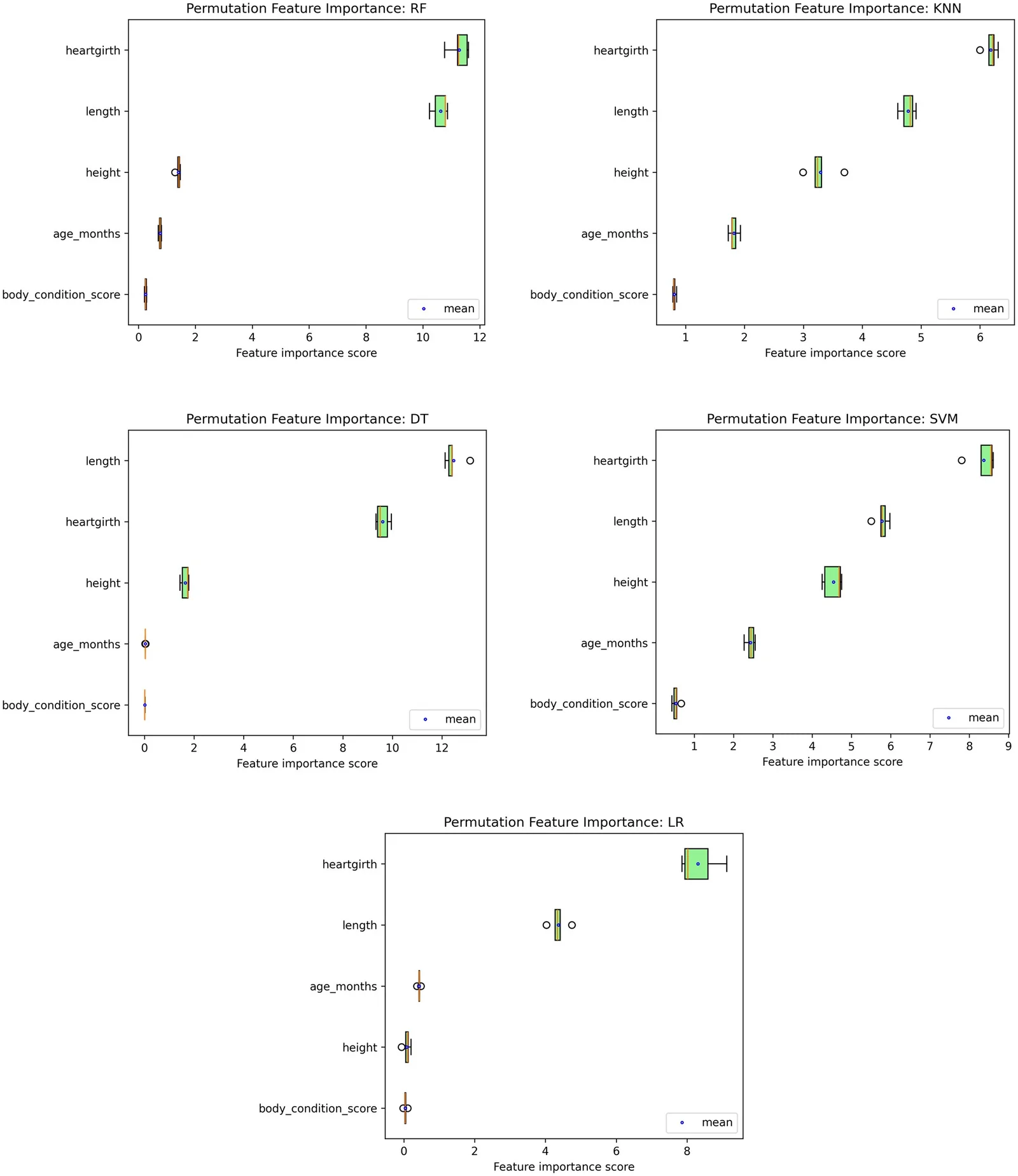
Case study 1—Feature importance for the five machine learning algorithms applied on the training set. Each plot depicts the feature importance score representing the change in the model’s performance measure (MAE) after randomly shuffling the values of each feature. The five input features are the age (months), heart girth, height, length, body condition score and the body weight of pigs made available by Marshall et al. (2023).

***Feature importance analysis:*** To better understand the influence of each input variable on body weight predictions, we used permutation feature importance across all five algorithms. As shown in Figure 9, heart girth and body length consistently emerged as the most important predictors, while height, age, and body condition score contributed far less to the models’ predictive performance. These findings align with prior research on swine body weight estimation, which also identified heart girth and length, often modeled with linear or quadratic terms, as key predictors (Groesbeck et al., 2002; Sungirai et al., 2014; Marshall et al., 2023; Thapar et al., 2023).

### Case study 2: Dry matter intake prediction in hair sheep

Modeling dry matter intake (**DMI**) in livestock is essential for optimizing nutritional management, improving feed efficiency, and supporting animal health and productivity. Accurate DMI predictions allow producers to formulate balanced diets, reduce feed waste, and enhance economic returns. In the context of hair sheep, which are increasingly valued for their adaptability and low-input requirements, modeling DMI is particularly important due to their diverse genetic backgrounds, variable grazing behaviors, and sensitivity to environmental conditions. Understanding and predicting DMI in hair sheep helps tailor feeding strategies that align with their unique physiological and production traits, contributing to more sustainable and efficient sheep production systems.

This case study uses data from a meta-analysis study published by de Oliveira et al. (2020). The data was collected from 61 studies, comprising 413 experimental units, to investigate dry matter intake (DMI) in hair sheep. The studies were sourced from public databases and Brazilian theses using keywords such as “dry matter intake,” “lambs,” and “sheep in tropical environments.” All studies published between 2002 and 2019 focused on hair sheep in the growing and finishing phases under tropical conditions and included relevant quantitative data such as neutral detergent fiber (NDF) levels, body weight, average daily gain (ADG), and fiber intake. Most studies focused on dietary changes and feed additives. Data were extracted independently, and additional variables like fiber digestibility were used to estimate rumen fill, accounting for intake differences between smaller and larger lambs due to variations in body weight and rumen capacity.

Data preparation: The raw file made available by de Oliveira et al. (2020) has been pre-manually processed such that only six columns are kept (breed, sex, NDF, ADG, body weight, and DMI), and five records with missing values were deleted. The file is imported into Python, where duplicate records are sought and removed (none present). Outliers are identified and excluded using the Z-score method, which filters out records that fall more than four standard deviations from the mean. The resulting cleaned dataset, containing 408 records, is saved to a new file for use in subsequent analyses.

**Figure 10.**
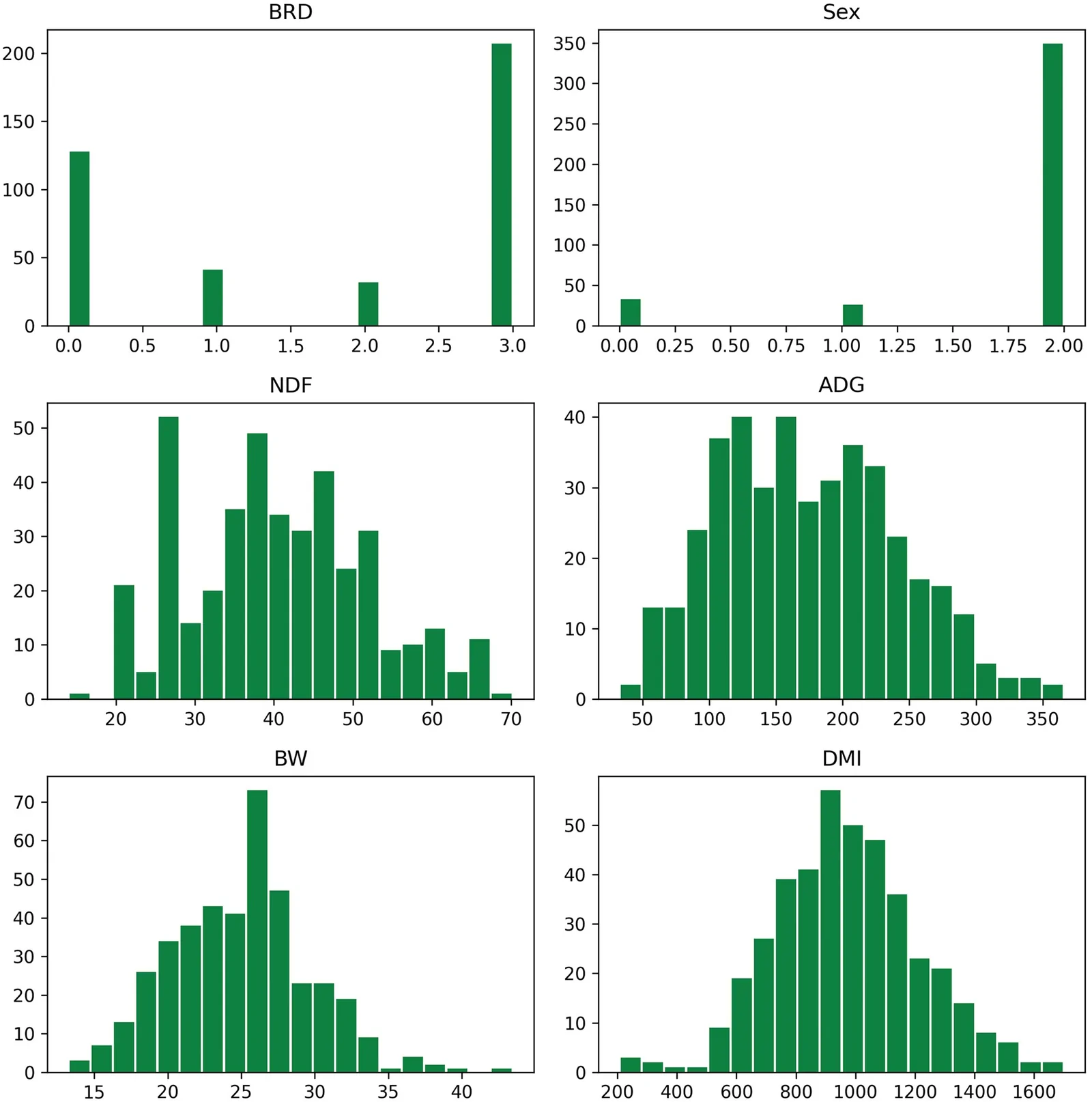
Case study 2—Variable distributions. The histograms represent the distribution of breed, sex (0 = castrated male, 1 = female, 2 = non-castrated male), NDF, ADG, body weight and dry matter intake (kg) collected from 408 hair sheep published by de Oliveira et al. (2020).

**Figure 11.**
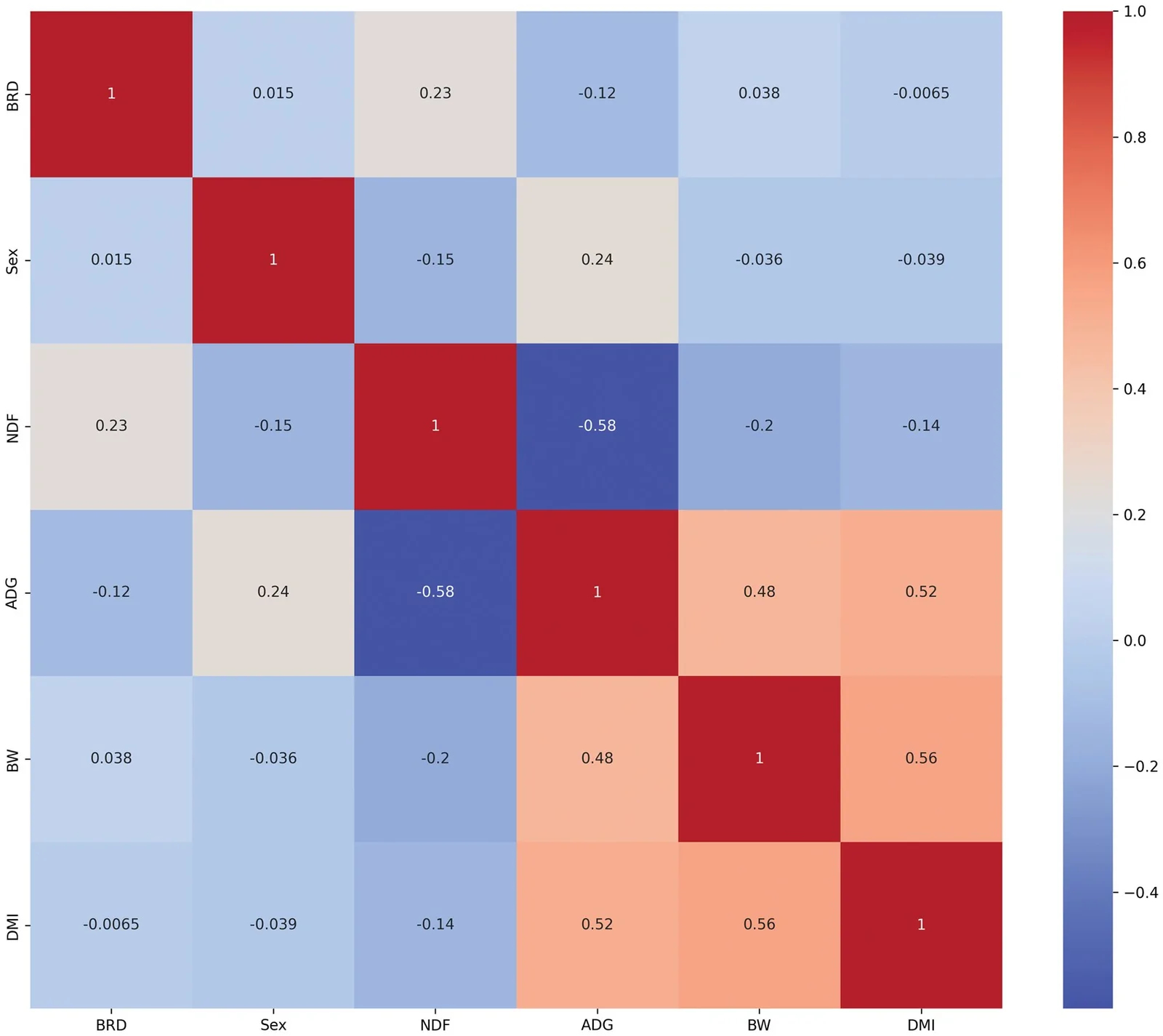
Case study 2—Pearson product-moment correlation scores for pairs of variables. The figure depicts Pearson correlation scores among all pairs of variables representing the breed, sex, NDF, ADG, body weight and dry matter intake of 408 hair sheep used in a study by de Oliveira et al. (2020).

Data visualization: The distributions of individual variables are illustrated using histograms (Figure 10), while Pearson product-moment correlation coefficients are calculated for all pairs of variables and displayed in a correlation matrix plot (Figure 11). Body weight and ADG exhibit a moderate correlation with DMI, whereas the remaining variables display either weak or no correlation.

***Model development and evaluation:*** The training dataset consists of 326 records, representing 80% of the total data, and includes five input variables—breed, sex, height, NDF, ADG, and body weight—used to predict the target variable, DMI in grams. Five widely used machine learning algorithms are implemented: KNN, LR, DT, RF, and SVM. Model performance is assessed using repeated 5-fold cross-validation with three repetitions and evaluation on an independent test set comprising the remaining 20% of the data (82 records). In this case, performance is measured using MAE, although additional evaluation measures can also be applied.

***Hyperparameter optimization:*** Each machine learning model includes user-defined parameters, known as hyperparameters, which can be tuned to improve performance. In this example, a grid search approach evaluating all possible parameter combinations is used to identify the optimal settings. A complete list of the hyperparameter values tested and their best-performing values is provided in Table 3.

**Table 3.** Case study 2—List of hyperparameters, ranges of values and best settings for five ML algorithms

Algorithm hyperparameters	Range of values	Best value
*Linear Regression*		
**fit_intercept**	[True, False]	True
*K-Nearest Neighbour*		
**n_neighbors**	[1 : 10]—integers	3
*Decision Tree*		
**criterion**	[‘friedman_mse’, ‘absolute_error’, ‘poisson’, ‘squared_error’]	‘squared_error’
**max_depth**	[1 : 9]—integers	6
*Support Vector Machine*		
**C**	[0 : 1.6]—increments of 0.2	1.41
*Random Forest*		
**n_estimators**	[20, 50, 100, 150, 200]	200
**max_depth**	[2 : 10]—integers	10

The best values for each hyperparameters were obtained using a grid search approach where all possible hyperparameter value combinations are attempted for each algorithm. The mean absolute error was used in this optimization process.

**Figure 12.**
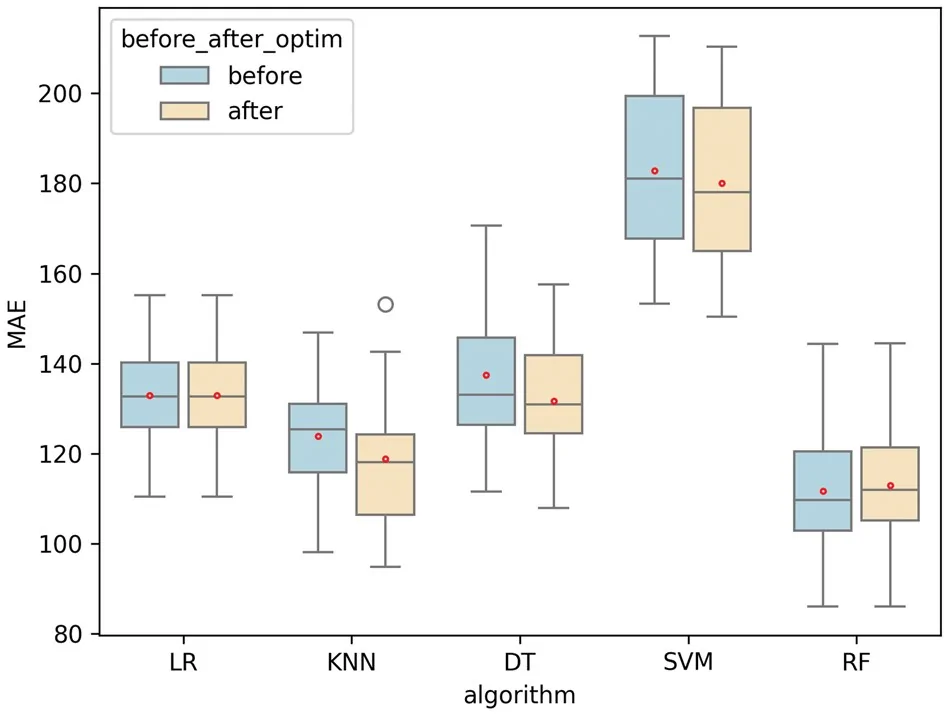
Case study 2—Cross-validation algorithm performance before and after hyperparameter optimization. The figure depicts box plots representing the MAE results for DMI predictions obtained with five ML algorithms (Linear Regression, K-Nearest Neighbour, Decision Tree, Support Vector Machine and Random Forest) applied on a dataset published by de Oliveira et al. (2020).

**Figure 13.**
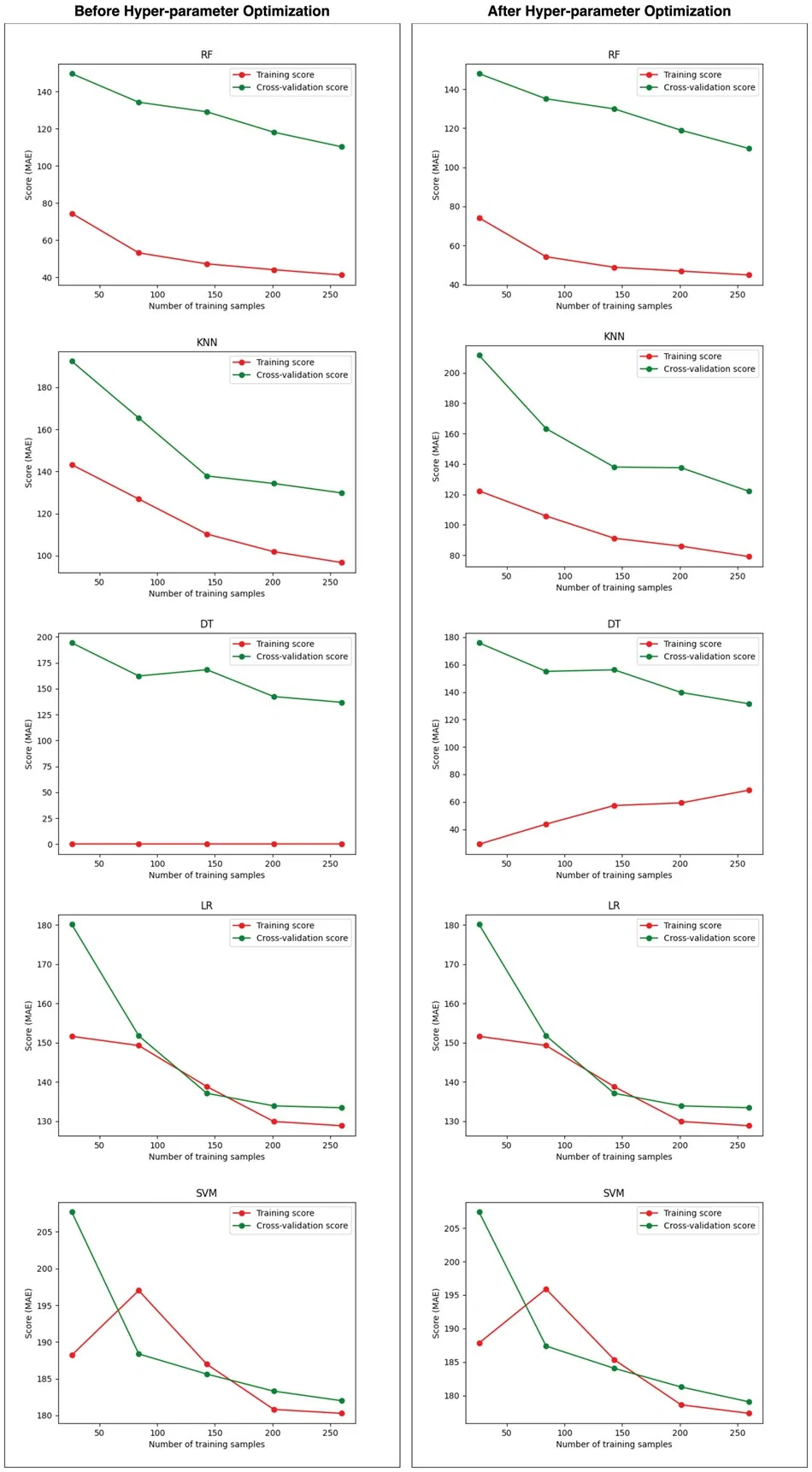
Case study 2—Learning curves depicting fitting performance for five machine learning algorithms predicting DMI. The five machine learning models are Linear Regression, K-Nearest Neighbour, Decision Tree, Support Vector Machine and Random Forest.

***Prediction results and fitting analysis*** *:* Among the five algorithms evaluated, Random Forest delivered the best performance, achieving the lowest MAE during cross-validation, with 111.64 g/day before HPO and 112.86 g/day after HPO, and an MAE of 128.82 g/day on the test set. It was followed by KNN, DT, and LR, while SVM performed the poorest, with an MAE of 187.91 g/day. Detailed performance measures are presented in Table 4 and Figure 12. Overfitting levels were visually assessed based on the gap between training and validation curves using MAE-based thresholds: No (0–2 MAE), Low (2–4 MAE), Medium (4–6 MAE), and High (>6 MAE). Learning curves (Figure 13) were used to assess model generalization, revealing medium to high overfitting in the top-performing models, whereas Support Vector Regression and Linear Regression showed minimal signs of overfitting. Hyperparameter tuning slightly improved the Decision Tree model’s performance and generalization.

**Table 4.** Case study 2—Algorithms cross-validation and testing performance

Algorithm	Cross-validation MAE (std) before HPO	Overfitting	Cross-validation MAE (std) after HPO	Overfitting	Testing MAE after HPO
**Random Forest**	111.64 (16.02)	High	112.86 (15.15)	High	128.82
**K-Nearest Neighbour**	123.86 (12.86)	Medium	118.86 (15.56)	Medium	131.49
**Decision Tree**	137.37 (15.97)	High	131.69 (13.68)	Medium	131.90
**Linear Regression**	132.88 (12.64)	No	132.88 (12.64)	No	149.99
**Support Vector Machine**	182.87 (18.77)	No	180.06 (18.74)	No	187.91

For each of the 5 algorithms we report the validation MAE (g/day) and overfitting results before and after hyperparameter optimization and the testing MAE results. HPO, hyperparameter optimization; MAE, means absolute error; std, standard deviation.

**Figure 14.**
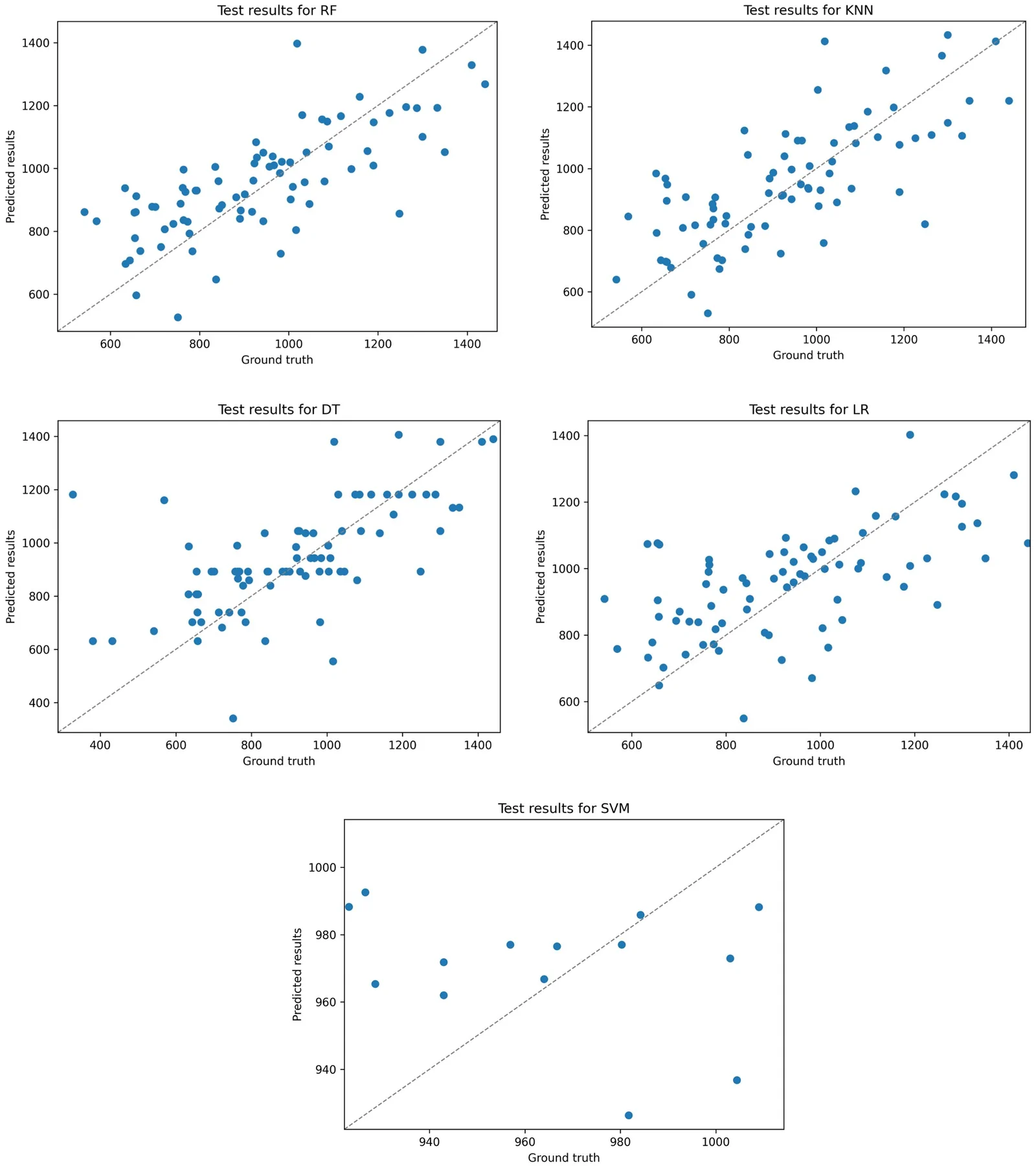
Case study 2—Scatter plots representing the predictions of the five machine learning algorithms on the testing set. The five machine learning models are Linear Regression, K-Nearest Neighbour, Decision Tree, Support Vector Machine and Random Forest. Each plot represents predicted versus ground truth dry matter intake of 82 hair sheep from a study by de Oliveira et al. (2020).

**Figure 15.**
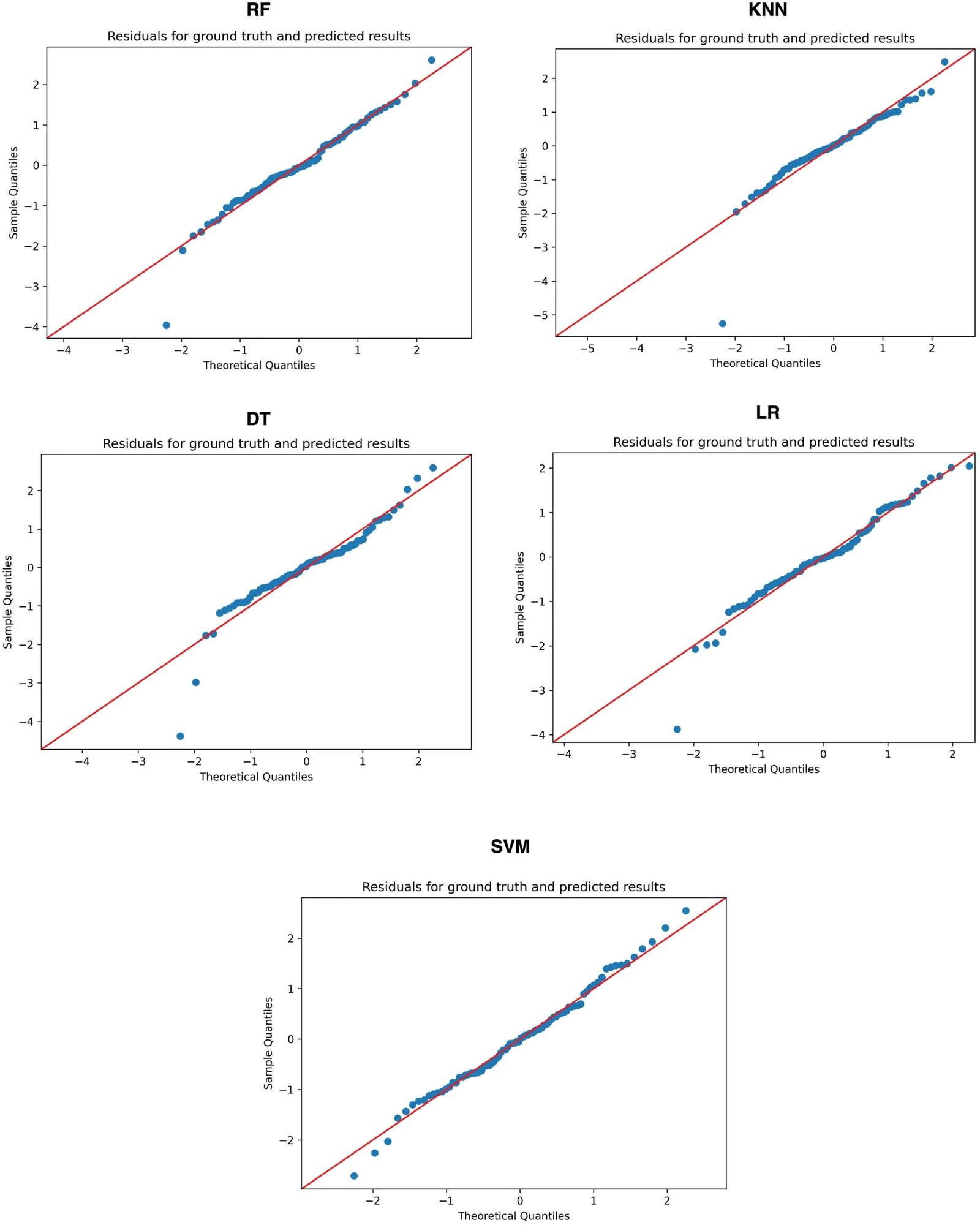
Case study 2—Quantile-Quantile plots for errors resulting from the five machine learning models applied on the testing set. The five machine learning models are Linear Regression, K-Nearest Neighbour, Decision Tree, Support Vector Machine and Random Forest. In each plot, the red line represents f(x) = x, which indicates where points would fall if the data followed the theoretical distribution (t-distribution) exactly. The blue dots represent the DMI residual values. The DMI values were predicted based on a test set including 82 hair sheep from a study by de Oliveira et al. (2020).

Scatter plots and quantile-quantile (Q-Q) plots were generated to investigate predictive performance and error distribution further (Figures 14 and 15). The top four models demonstrated low to moderate predictive performance for DMI, while SVM struggled with this dataset. However, the Q-Q plots indicated minimal deviation from the expected normal distribution of residuals, suggesting that while overall accuracy was limited, the models maintained reasonable generalization across the DMI range. These results highlight the importance of thorough fit diagnostics, even when using established algorithms with strong theoretical underpinnings.

**Figure 16.**
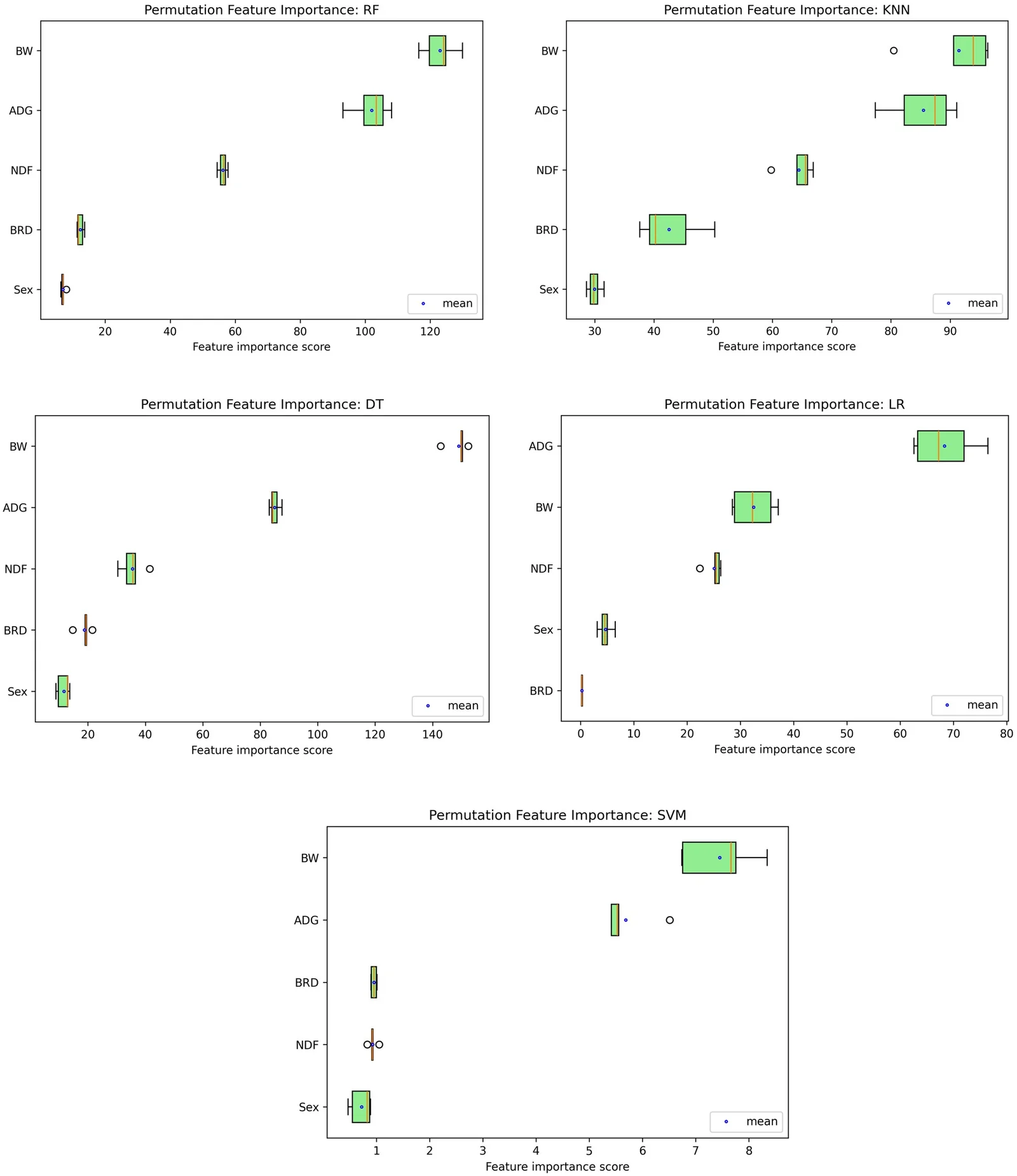
Case study 2—Feature importance for the five machine learning algorithms. Each plot depicts the feature importance score representing the change in the model’s performance measure (MAE) after randomly shuffling the values of each feature. The five input features are breed, sex, NDF, ADG, body weight and dry matter intake of hair sheep made available by de Oliveira et al. (2020).

***Feature importance analysis:*** To better understand the influence of each input variable on dry matter intake predictions, we used permutation feature importance across all five algorithms. As shown in Figure 16, body weight and ADG consistently emerged as the most important predictors, while NDF, breed, and especially sex contributed far less to the models’ predictive performance.

Body weight is the most important and reliable predictor of DMI. Heavier animals have greater maintenance energy requirements and larger rumen capacity, increasing dry matter intake. Prediction models such as those from the NRC and CNCPS routinely include BW or metabolic BW (${BW}^{0.75}$) as key inputs due to their physiological relevance (Soest, 1994; Tedeschi, 2006). ADG, which reflects growth rate and nutrient demand, also plays a significant role (Cannas et al., 2004; Castro-Montoya and Dickhoefer, 2020). Animals with higher ADG typically consume more to support tissue accretion, making it a valuable predictor when modeling intake in growing animals. NDF, representing the fiber content of the diet, influences DMI through its effects on rumen fill and digestion kinetics (Mertens, 1994; Fox et al., 2004). High-NDF diets are bulkier and slower to digest, which can physically limit intake, especially in forages with low digestibility. Consequently, NDF is a crucial dietary variable when modeling intake capacity, particularly in ruminants. The breed also affects DMI, as genetic differences influence size, metabolism, and feed efficiency (Goetsch et al., 2011). For instance, hair sheep often consume less than wool breeds due to their smaller frame and lower maintenance requirements. Lastly, sex has a modest but notable effect on DMI (Jaborek et al., 2018). Males generally eat more than females due to differences in growth rate and hormonal influences, though this effect is less pronounced than other factors like BW or ADG.

In summary, the application of our computational pipeline suggests that effective modeling of DMI in livestock, especially in species like hair sheep, should prioritize variables such as body weight, ADG, and NDF while considering breed and sex as secondary predictors. Incorporating these variables can improve prediction performance and help design feeding systems tailored to the specific nutritional needs of diverse animal populations.

## Conclusions

This study highlights the value of using open-source machine learning pipelines to streamline predictive modeling in animal science. Through a practical example focused on swine body weight prediction, we demonstrate that machine learning models like Random Forest and K-Nearest Neighbors can effectively utilize simple morphometric inputs to produce accurate, interpretable predictions. The open-source nature of the pipeline allows for transparency, reproducibility, and easy adaptation, making it a valuable tool for researchers, educators, and students alike. Importantly, such tools serve as practical educational platforms, helping bridge the gap between traditional animal science training and the growing demand for data-driven decision-making in precision livestock farming. Echoing the recommendations of Brennan et al. (2023), this work illustrates how openly shared computational workflows can enhance digital literacy, improve model transparency, and empower animal scientists to engage confidently with big data and predictive modeling.

## Challenges and Future Directions

Future work should focus on expanding the scope and impact of machine learning applications in animal science by integrating diverse data types, such as sensor, behavioral, genomic, or environmental data, into in-line and real-time ML predictive pipelines. Such multi-modal approaches enable earlier detection of health and performance issues while supporting more robust and actionable decision-making. For instance, Ferreira et al. (2024) demonstrated how combining heterogeneous data streams in a cloud-based machine learning framework improved the early detection of metabolic disorders in dairy cows, underscoring the potential of integrated, real-time solutions to transform livestock management. This would enhance the depth of analysis and support broader applications.

At the same time, it is important to recognize that prediction and actionable insight are not equivalent. While predictive models can identify at-risk animals or forecast outcomes, causal inference provides the foundation for efficient intervention by revealing which factors truly drive changes in livestock systems. The interpretation of regression models differs significantly depending on whether data arise from randomized trials or observational studies, and overlooking this distinction risks drawing misleading conclusions. Even with observational data, however, a range of regression-based causal inference frameworks exists to help disentangle association from causation (Bello et al., 2018; Sargeant et al., 2024). Incorporating such approaches into ML pipelines could substantially increase their utility, moving from systems that only flag potential issues to those that can also inform intervention strategies.

Additionally, efforts should be directed at testing the robustness and generalizability of these models through repeated train-test sampling and external validation across various farms or regions. Interactive learning modules, such as embedding open-source code in platforms like Jupyter Notebooks or R Shiny apps, can facilitate hands-on learning and practical applications for students and researchers. Promoting collaborative code development via platforms like GitHub will allow for continuous improvement and adaptation of the pipeline to different livestock contexts. Furthermore, incorporating model explainability tools, such as SHAP or LIME, will ensure a better understanding of predictions, particularly for applications influencing animal welfare or resource management. By embracing open-source development practices, this approach will drive collaboration, innovation, and skill-building, empowering the next generation of researchers to harness big data effectively in animal science.

## Data Availability

The dataset and accompanying Python code are openly accessible in the Supplementary Materials and at: https://github.com/dtulpan/ML_regression_pipeline_education.

## Abbreviations:

DL: Deep Learning
DT: Decision Tree
FN: False Negatives
FP: False Positives
IoT: Internet of Things
KNN: K-Nearest Neighbors
LDA: Linear ­Discriminant Analysis (LDA)
LightGBM: Light Gradient-Boosting Machine
LR: Linear Regression
ML: Machine Learning
PCA: Principal Component Analysis
RF: Random Forest
RFE: Recursive Feature Elimination
ROC: Receiver Operating Characteristic
SVM: Support Vector Machines
TN: True Negatives
TP: True Positives
XGBoost: eXtreme Gradient Boost
