# Correction to: ASAS-NANP Symposium: mathematical modeling in animal nutrition: construction of supervised machine learning regression pipelines for livestock data modeling: a case studys

**DOI:** 10.1093/jas/skag228

**Published:** 2026-09-02

This is a correction to: Dan Tulpan, Luis O Tedeschi, Hector Menendez, Ricardo Augusto M Vieira, ASAS-NANP Symposium: mathematical modeling in animal nutrition: construction of supervised machine learning regression pipelines for livestock data modeling: a case studys, *Journal of Animal Science*, Volume 104, 2026, skaf444, https://doi.org/10.1093/jas/skaf444

In the originally published version of this manuscript, four workflow processes in Figure 1 were presented out of order. The original order was: ‘Model training’, ‘Model initialization’, ‘Data scalling’, ‘Data Splitting’. The correct order should be ‘Model initialization’, ‘Data splitting’, ‘Data scalling’, ‘Model training’.

The corrected Figure 1 is reproduced here:

**Figure:**
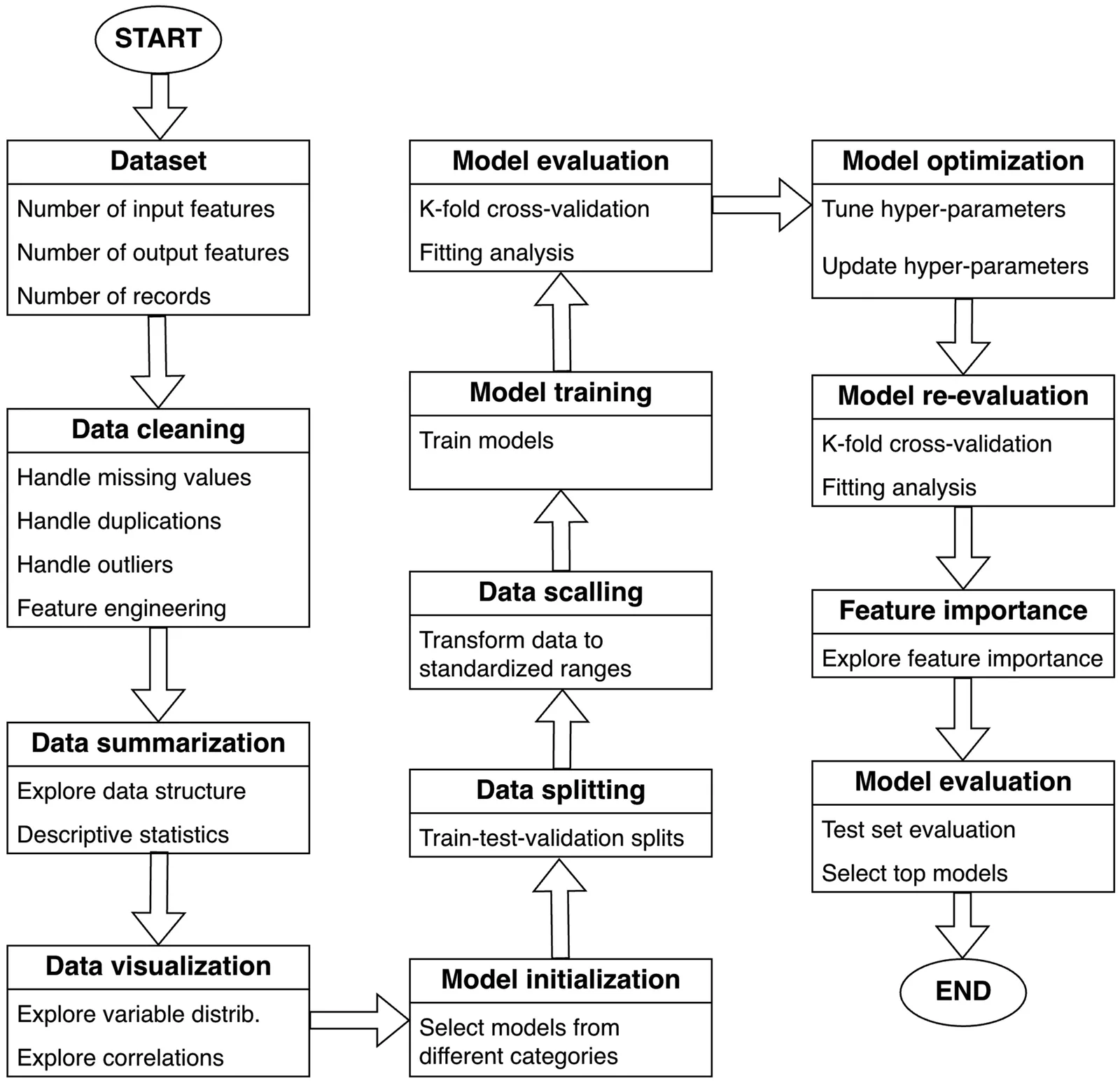


This error has been corrected.

